# Management of bleeding and coagulopathy following major trauma: an updated European guideline

**DOI:** 10.1186/cc12685

**Published:** 2013-04-19

**Authors:** Donat R Spahn, Bertil Bouillon, Vladimir Cerny, Timothy J Coats, Jacques Duranteau, Enrique Fernández-Mondéjar, Daniela Filipescu, Beverley J Hunt, Radko Komadina, Giuseppe Nardi, Edmund Neugebauer, Yves Ozier, Louis Riddez, Arthur Schultz, Jean-Louis Vincent, Rolf Rossaint

**Affiliations:** 1 Institute of Anaesthesiology, University Hospital Zurich, Rämistrasse 100, CH-8091 Zurich, Switzerland; 2 Department of Trauma and Orthopaedic Surgery, University of Witten/Herdecke, Cologne-Merheim Medical Centre, Ostmerheimerstrasse 200, D-51109 Cologne, Germany; 3 Faculty of Medicine in Hradec Králové, Department of Anaesthesiology and Intensive Care Medicine, University Hospital Hradec Králové, CZ-50005 Hradec Králové, Czech Republic; 4 Dalhousie University, Department of Anesthesia, Pain Management and Perioperative Medicine, Halifax, NS B3H 4R2, Canada; 5 Accident and Emergency Department, University of Leicester, Infirmary Square, Leicester LE1 5WW, UK; 6 Department of Anaesthesia and Intensive Care, University of Paris XI, Faculté de Médecine Paris-Sud, 63 rue Gabriel Péri, F-94276 Le Kremlin-Bicêtre, France; 7 Department of Emergency and Critical Care Medicine, University Hospital Virgen de las Nieves, ctra de Jaén s/n, E-18013 Granada, Spain; 8 Department of Cardiac Anaesthesia and Intensive Care, C. C. Iliescu Emergency Institute of Cardiovascular Diseases, Sos Fundeni 256-258, RO-022328 Bucharest, Romania; 9 Guy's and St Thomas' Foundation Trust, Westminster Bridge Road, London, SE1 7EH, UK; 10 Department of Traumatology, General and Teaching Hospital Celje, SI-3000 Celje, Slovenia; 11 Shock and Trauma Centre, S. Camillo Hospital, Viale Gianicolense 87, I-00152 Rome, Italy; 12 Institute for Research in Operative Medicine (IFOM), Witten/Herdecke University, Campus Cologne, Ostmerheimerstrasse 200, D-51109 Cologne, Germany; 13 Division of Anaesthesia, Intensive Care and Emergency Medicine, Brest University Hospital, Boulevard Tanguy Prigent, F-29200 Brest, France; 14 Department of Surgery and Trauma, Karolinska University Hospital, S-171 76 Solna, Sweden; 15 Ludwig-Boltzmann-Institute for Experimental and Clinical Traumatology, Lorenz Boehler Trauma Centre, Donaueschingenstrasse 13, A-1200 Vienna, Austria; 16 Department of Intensive Care, Erasme University Hospital, Université Libre de Bruxelles, Route de Lennik 808, B-1070 Brussels, Belgium; 17 Department of Anaesthesiology, University Hospital Aachen, RWTH Aachen University, Pauwelsstrasse 30, D-52074 Aachen, Germany

## Abstract

**Introduction:**

Evidence-based recommendations are needed to guide the acute management of the bleeding trauma patient. When these recommendations are implemented patient outcomes may be improved.

**Methods:**

The multidisciplinary Task Force for Advanced Bleeding Care in Trauma was formed in 2005 with the aim of developing a guideline for the management of bleeding following severe injury. This document represents an updated version of the guideline published by the group in 2007 and updated in 2010. Recommendations were formulated using a nominal group process, the Grading of Recommendations Assessment, Development and Evaluation (GRADE) hierarchy of evidence and based on a systematic review of published literature.

**Results:**

Key changes encompassed in this version of the guideline include new recommendations on the appropriate use of vasopressors and inotropic agents, and reflect an awareness of the growing number of patients in the population at large treated with antiplatelet agents and/or oral anticoagulants. The current guideline also includes recommendations and a discussion of thromboprophylactic strategies for all patients following traumatic injury. The most significant addition is a new section that discusses the need for every institution to develop, implement and adhere to an evidence-based clinical protocol to manage traumatically injured patients. The remaining recommendations have been re-evaluated and graded based on literature published since the last edition of the guideline. Consideration was also given to changes in clinical practice that have taken place during this time period as a result of both new evidence and changes in the general availability of relevant agents and technologies.

**Conclusions:**

A comprehensive, multidisciplinary approach to trauma care and mechanisms with which to ensure that established protocols are consistently implemented will ensure a uniform and high standard of care across Europe and beyond.

**Please see related letter by Morel *et al*http://ccforum.com/content/17/4/442**

## Introduction

Severe trauma is one of the major health care issues faced by modern society, resulting in the annual death of more than five million people worldwide, and this number is expected to increase to more than eight million by 2020 [1]. Uncontrolled post-traumatic bleeding is the leading cause of potentially preventable death among these patients [2,3]. Appropriate management of the massively bleeding trauma patient includes the early identification of bleeding sources followed by prompt measures to minimise blood loss, restore tissue perfusion and achieve haemodynamic stability.

**Figure 1:**
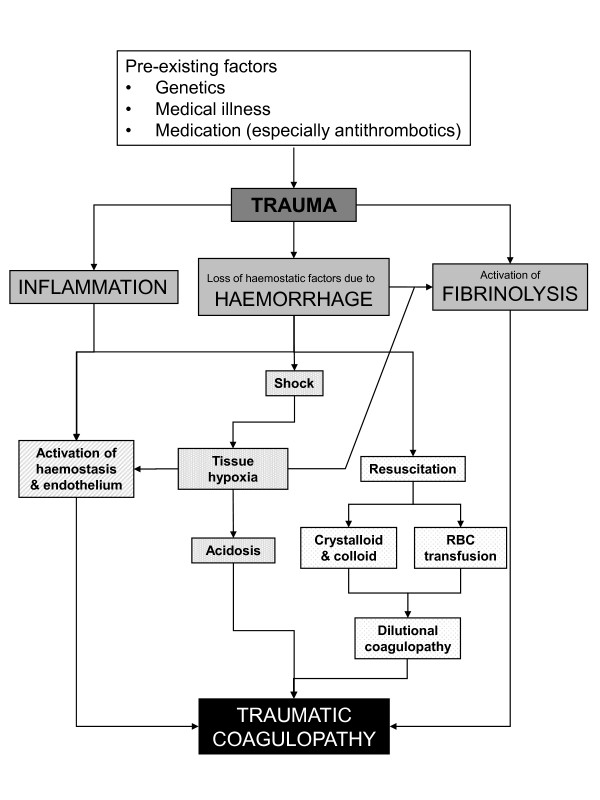
**Current concepts of pathogenesis of coagulopathy following traumatic injury**. Adapted from [9,10].

An awareness of the specific pathophysiology associated with bleeding following traumatic injury by treating physicians is essential. About one-third of all bleeding trauma patients present with a coagulopathy upon hospital admission [4-7]. This subset of patients has a significantly increased incidence of multiple organ failure and death compared to patients with similar injury patterns in the absence of a coagulopathy [4,5,7,8]. The early acute coagulopathy associated with traumatic injury has recently been recognised as a multifactorial primary condition that results from a combination of bleeding-induced shock, tissue injury-related thrombin-thrombomodulin-complex generation and the activation of anticoagulant and fibrinolytic pathways (Figure 1) [5-7,9-11]. Moreover, it has been shown that high circulating levels of syndecan-1, a marker of endothelial glycocalyx degradation, is associated with coagulopathy in trauma patients [12]. Different factors influence the severity of the coagulation disorder. On one hand, coagulopathy is influenced by environmental and therapeutic factors that result in or at least contribute to acidaemia, hypothermia, dilution, hypoperfusion and coagulation factor consumption [5,6,9,13-15]. On the other hand, this condition is modified by individual patient-related factors, including genetic background, co-morbidities, inflammation and medications, especially oral anticoagulants, and pre-hospital fluid administration [15-17]. A recent paper suggests that the severity of traumatic brain injury (TBI) represents a further individual patient-related factor that may contribute to acute traumatic coagulopathy [18]. A number of terms have been proposed to describe the condition, which is distinct from disseminated intravascular coagulation, including Acute Traumatic Coagulopathy [6,19], Early Coagulopathy of Trauma [7], Acute Coagulopathy of Trauma-Shock [9], Trauma-Induced Coagulopathy [20] and Trauma-Associated Coagulopathy [21].

This European guideline, originally published in 2007 [22] and updated in 2010 [23], represents a second update and is part of the European "*STOP the Bleeding Campaign*", an international initiative launched in 2013 to reduce morbidity and mortality associated with bleeding following traumatic injury. The campaign aims to support haemostatic resuscitation measures by providing clinical practice guidelines to ensure the early recognition and treatment of bleeding and traumatic coagulopathy. The acronym STOP stands for Search for patients at risk of coagulopathic bleeding, Treat bleeding and coagulopathy as soon as they develop, Observe the response to interventions and Prevent secondary bleeding and coagulopathy. As part of the campaign, this guideline should not only provide a better understanding of the pathophysiology of the severely bleeding patient following traumatic injury and treatment guidance for the clinician, but also highlight the areas in which further research is urgently required. The recommendations for in-hospital patient management have been adapted to reflect the evidence published during the last three years, a consideration of changes in clinical practice that have taken place during this period as well as new recommendations that reflect emerging areas of clinical relevance. Although the recommendations outline corridors for diagnosis and treatment, the author group believes that the greatest outcome improvement can currently be made through education and process adaptation. Therefore, our multidisciplinary group of European experts, including designated representatives from relevant professional societies, felt the need to define clinically relevant "bundles" for diagnosis and therapy, in order to facilitate the adaptation of the guiding principles to the local situation and implementation within each institution. We believe that adherence to the local management protocol should be assessed, and that such regular compliance assessments should be part of institutional quality management processes, and that personnel training to ensure compliance should be adapted accordingly. If followed, these clinical practice guidelines have the potential to ensure a uniform standard of care across Europe and beyond.

## Materials and methods

These recommendations were formulated and graded according to the Grading of Recommendations Assessment, Development and Evaluation (GRADE) hierarchy of evidence [24-26] summarised in Table 1. Comprehensive computer database literature searches were performed using the indexed online database MEDLINE/PubMed. Lists of cited literature within relevant articles were also screened. The primary intention of the review was to identify prospective randomised controlled trials (RCTs) and non-RCTs, existing systematic reviews and guidelines. In the absence of such evidence, case-control studies, observational studies and case reports were considered.

**Table 1:** Grading of recommendations after [24] (reprinted with permission)

Grade of Recommendation	Clarity of risk/benefit	Quality of supporting evidence	Implications
**1A**			
Strong recommendation, high-quality evidence	Benefits clearly outweigh risk and burdens, or vice versa	RCTs without important limitations or overwhelming evidence from observational studies	Strong recommendation, can apply to most patients in most circumstances without reservation
**1B**			
Strong recommendation, moderate-quality evidence	Benefits clearly outweigh risk and burdens, or vice versa	RCTs with important limitations (inconsistent results, methodological flaws, indirect or imprecise) or exceptionally strong evidence from observational studies	Strong recommendation, can apply to most patients in most circumstances without reservation
**1C**			
Strong recommendation, low-quality or very low-quality evidence	Benefits clearly outweigh risk and burdens, or vice versa	Observational studies or case series	Strong recommendation but may change when higher quality evidence becomes available
**2A**			
Weak recommendation, high-quality evidence	Benefits closely balanced with risks and burden	RCTs without important limitations or overwhelming evidence from observational studies	Weak recommendation, best action may differ depending on circumstances or patients' or societal values
**2B**			
Weak recommendation, moderate-quality evidence	Benefits closely balanced with risks and burden	RCTs with important limitations (inconsistent results, methodological flaws, indirect or imprecise) or exceptionally strong evidence from observational studies	Weak recommendation, best action may differ depending on circumstances or patients' or societal values
**2C**			
Weak recommendation, Low-quality or very low-quality evidence	Uncertainty in the estimates of benefits, risks and burden; benefits, risk and burden may be closely balanced	Observational studies or case series	Very weak recommendation; other alternatives may be equally reasonable

Boolean operators and Medical Subject Heading (MeSH) thesaurus keywords were applied as a standardised use of language to unify differences in terminology into single concepts. Appropriate MeSH headings and subheadings for each question were selected and modified based on search results. The scientific questions posed that led to each recommendation and the MeSH headings applied to each search are listed in Additional file 1. Searches were limited to English-language abstracts and human studies; gender and age were not limited. The time period was limited to the past three years for questions addressed in the 2010 version of the guideline. A time period limit of 10 years was applied to new searches yielding more than 500 hits; otherwise no time-period limits were imposed. Abstracts from original publications were screened for relevance and full publications evaluated where appropriate. Some additional citations that were published after the literature search cut-off for the guideline document are listed in Additional file 2; these publications were not selected according to a comprehensive search strategy, but represent work with sufficient relevance to the guideline that inclusion was requested by one or more of the endorsing professional societies as part of the guideline review and endorsement process.

Selection of the scientific enquiries to be addressed in the guideline, screening and grading of the literature to be included and formulation of specific recommendations were performed by members of the Task Force for Advanced Bleeding Care in Trauma, a multidisciplinary, pan-European group of experts with specialties in surgery, anaesthesia, emergency medicine, intensive care medicine and haematology. The core group was formed in 2004 to produce educational material on the care of the bleeding trauma patient on which an update (2006) and subsequent review article [27] were based. The task force consisted of the core group, additional experts in haematology and guideline development, and representatives of relevant European professional societies, including the European Society of Anaesthesiology, the European Society of Intensive Care Medicine, the European Shock Society, the European Society of Trauma and Emergency Surgery and the European Society for Emergency Medicine. The European Hematology Association declined the invitation to designate a representative to join the task force. As part of the guideline development process that led to the 2007 guideline [22], task force members participated in a workshop on the critical appraisal of medical literature. An updated version of the guideline was published in 2010 [23]. The nominal group process for the current update of the guideline included several remote (telephone and web-based) meetings and one face-to-face meeting supplemented by electronic communication. The guideline development group participated in a web conference in January 2012 to define the scientific questions to be addressed in the guideline. Selection, screening and grading of the literature and formulation of recommendations were accomplished in subcommittee groups consisting of two to five members via electronic or telephone communication. After distribution of the recommendations to the entire group, a face-to-face meeting of the task force was held in April 2012 with the aim of reaching a consensus on the draft recommendations from each subcommittee. After final refinement of the rationale for each recommendation and the complete manuscript, the updated document was approved by the endorsing organisations between September 2012 and January 2013. An updated version of the guideline is anticipated in due time.

In the GRADE system for assessing each recommendation, the letter attached to the grade of recommendation reflects the degree of literature support for the recommendation, whereas the number indicates the level of support for the recommendation assigned by the committee of experts. Recommendations are grouped by category and somewhat chronologically in the treatment decision-making process, but not by priority or hierarchy.

## Results

### I. Initial resuscitation and prevention of further bleeding

#### Minimal elapsed time

##### Recommendation 1

We recommend that the time elapsed between injury and operation be minimised for patients in need of urgent surgical bleeding control. (Grade 1A)

###### Rationale

Trauma patients in need of emergency surgery for ongoing hemorrhage have increased survival if the elapsed time between the traumatic injury and admission to the operating theatre is minimised. More than 50% of all trauma patients with a fatal outcome die within 24 h of injury [3]. Despite a lack of evidence from prospective RCTs, well-designed retrospective studies provide evidence for early surgical intervention in patients with traumatic haemorrhagic shock [28-30]. In addition, studies that analyse trauma systems indirectly emphasise the importance of minimising the time between admission and surgical bleeding control in patients with traumatic haemorrhagic shock [31,32]. At present, the evidence base for the impact of the implementation of the Advanced Trauma Life Support (ATLS) protocol on patient outcome is very poor, because the available literature focuses primarily on the effectiveness of ATLS as an educational tool [33]. Future studies are needed to define the impact of the ATLS programme within trauma systems at the hospital and health system level in terms of controlled before-and-after implementation designed to assess post-injury mortality as the primary outcome parameter.

#### Tourniquet use

##### Recommendation 2

We recommend adjunct tourniquet use to stop life-threatening bleeding from open extremity injuries in the pre-surgical setting. (Grade 1B)

###### Rationale

When uncontrolled arterial bleeding occurs from mangled extremity injuries, including penetrating or blast injuries or traumatic amputations, a tourniquet represents a simple and efficient method with which to acutely control hemorrhage [34-38]. Tourniquet application has become standard of care for the control of severe hemorrhage following military combat injuries, and several publications report the effectiveness of tourniquets in this specific setting [34-37,39]. A study of volunteers showed that any tourniquet device presently on the market works efficiently [38]. The study also showed that 'pressure point control' was ineffective because collateral circulation was observed within seconds. Tourniquet-induced pain was not an important consideration. Tourniquets should be left in place until surgical control of bleeding is achieved [35,37]; however, this time span should be kept as short as possible. Improper or prolonged placement of a tourniquet can lead to complications, such as nerve paralysis and limb ischemia [40]; however, these effects are rare [39]. Some publications suggest a maximum time of application of two hours [40]. Reports from military settings report cases in which tourniquets have remained in place for up to six hours with survival of the extremity [35]. Much discussion has been generated recently regarding the translation of this evidence to civilian practice as there is no published evidence. Bleeding from most civilian wounds can be controlled by local pressure; however, there are case reports of effective bleeding control by the use of a tourniquet in civilian mangled extremity injury.

#### Ventilation

##### Recommendation 3

We recommend initial normoventilation of trauma patients if there are no signs of imminent cerebral herniation. (Grade 1C)

###### Rationale

Ventilation can affect the outcome of severe trauma patients. There is a tendency for rescue personnel to hyperventilate patients during resuscitation [41,42], and hyperventilated trauma patients appear to have increased mortality when compared with non-hyperventilated patients [42]. For the purpose of this discussion, the target arterial PaCO_2 _should be 5.0 to 5.5 kPa.

A high percentage of severely injured patients with ongoing bleeding have TBI. Relevant experimental and clinical data have shown that routine hyperventilation is an important contributor to adverse outcomes in head-injured patients; however, the effect of hyperventilation on outcome in patients with severe trauma but no TBI is still a matter of debate. A low PaCO_2 _on admission to the emergency room is associated with a worse outcome in trauma patients with TBI [43-46].

There are several potential mechanisms for the adverse effects of hyperventilation and hypocapnia, including increased vasoconstriction with decreased cerebral blood flow and impaired tissue perfusion. In the setting of absolute or relative hypovolaemia, an excessive rate of positive-pressure ventilation may further compromise venous return and produce hypotension and even cardiovascular collapse [44,45]. It has also been shown that cerebral tissue lactic acidosis occurs almost immediately after induction of hypocapnia in children and adults with TBI and haemorrhagic shock [47]. In addition, an even modest level of hypocapnia (<27 mmHg) may result in neuronal depolarisation with glutamate release and extension of the primary injury via apoptosis [48].

Ventilation with low tidal volume (<6 ml/kg) is recommended in patients with acute lung injury. In patients with normal lung function, the evidence is scarce, but some observational studies show that the use of a large tidal volume is an important risk factor for the development of lung injury [49,50]. The injurious effect of high tidal volume may be initiated very early. Randomised studies demonstrate that short-term ventilation (<5 h) with high tidal volume (12 ml/kg) without positive end-expiratory pressure (PEEP) may promote pulmonary inflammation and alveolar coagulation in patients with normal lung function [51]. Although more studies are needed, the early use of protective ventilation with low tidal volume and moderate PEEP is recommended, particularly in bleeding trauma patients at risk of acute lung injury.

### II. Diagnosis and monitoring of bleeding

#### Initial assessment

##### Recommendation 4

We recommend that the physician clinically assess the extent of traumatic hemorrhage using a combination of patient physiology, anatomical injury pattern, mechanism of injury and the patient's response to initial resuscitation. (Grade 1C)

###### Rationale

Visual estimation of the amount of blood loss at the scene of trauma can provide important information, but may be highly influenced by physiologic parameters suggesting normo or hypovolaemia [52]. The mechanism of injury represents an important screening tool with which to identify patients at risk for significant traumatic hemorrhage. For example, the American College of Surgeons defined a threshold of 6 m (20 ft) as a "critical falling height" associated with major injuries [53]. Further critical mechanisms include blunt versus penetrating trauma, high energy deceleration impact, low velocity versus high velocity gunshot injuries and so on. The mechanism of injury in conjunction with injury severity, as defined by trauma scoring systems, and the patient's physiological presentation and response to resuscitation should further guide the decision to initiate early surgical bleeding control as outlined in the ATLS protocol [54-57]. Table 2 summarises estimated blood loss based on initial presentation according to the ATLS classification system. Although the ATLS classification is a useful guide in haemorrhagic shock, a recent retrospective analysis of the validity of this classification system showed that increasing blood loss produces an increase in heart rate and decrease in blood pressure, but to a lesser degree than suggested by the ATLS classification. In addition, there are no significant changes in respiratory rate or in conscience level with bleeding [58]. Table 3 characterises the three types of response to initial fluid resuscitation, whereby the transient responders and the non-responders are candidates for immediate surgical bleeding control.

**Table 2:** ATLS classification of blood loss* based on initial patient presentation

	Class I	Class II	Class III	Class IV
Blood loss (ml)	Up to 750	750 to 1,500	1,500 to 2,000	>2,000
Blood loss (% blood volume)	Up to 15%	15% to 30%	30% to 40%	>40%
Pulse rate (bpm)	<100	100 to 120	120 to 140	>140
Systolic blood pressure	Normal	Normal	Decreased	Decreased
Pulse pressure (mmHg)	Normal or increased	Decreased	Decreased	Decreased
Respiratory rate	14 to 20	20 to 30	30 to 40	>35
Urine output (ml/h)	>30	20 to 30	5 to 15	Negligible
CNS/mental status	Slightly anxious	Mildly anxious	Anxious, confused	Confused, lethargic
Initial fluid replacement	Crystalloid	Crystalloid	Crystalloid and blood	Crystalloid and blood

Table reprinted with permission from the American College of Surgeons [57]. *for a 70 kg man.

**Table 3:** ATLS responses to initial fluid resuscitation

	Rapid response	Transient response	Minimal or no response
Vital signs	Return to normal	Transient improvement, recurrence of decreased blood pressure and increased heart rate	Remain abnormal
Estimated blood loss	Minimal (10% to 20%)	Moderate and ongoing (20% to 40%)	Severe (>40%)
Need for more crystalloid	Low	Low to moderate	Moderate as bridge to transfusion
Need for blood	Low	Moderate to high	Immediate
Blood preparation	Type and crossmatch	Type-specific	Emergency blood release
Need for operative intervention	Possibly	Likely	Highly likely
Early presence of surgeon	Yes	Yes	Yes

Table reprinted with permission from the American College of Surgeons [57]. *Isotonic crystalloid solution, 2,000 ml in adults; 20 ml/kg in children.

Specific scores to predict the risk of haemorrhagic shock may be useful to provide a prompt and appropriate treatment; however, its usefulness is still not optimal. Paladino *et al*. [59] analyzed the usefulness of the Shock Index (heart rate divided by systolic blood pressure) and found that this index may be useful in drawing attention to abnormal values, but that it is too insensitive to rule out disease and should not lower the suspicion of major injury. The TASH score (Trauma Associated Severe Hemorrhage) uses seven parameters (systolic blood pressure, haemoglobin (Hb), intra-abdominal fluid, complex long bone and/or pelvic fractures, heart rate, base excess and gender) to predict the probability of mass transfusion. Maegele *et al*. [60] retrospectively analysed a dataset of severely multiply-injured patients from the German Trauma Registry to confirm the validity of the TASH score to predict the individual probability of massive transfusion and, therefore, ongoing life-threatening hemorrhage. The TASH score has recently been re-validated with 5,834 patients from the same registry [61].

#### Immediate intervention

##### Recommendation 5

We recommend that patients presenting with haemorrhagic shock and an identified source of bleeding undergo an immediate bleeding control procedure unless initial resuscitation measures are successful. (Grade 1B)

###### Rationale

The source of bleeding may be immediately obvious, and penetrating injuries are more likely to require surgical bleeding control. In a retrospective study of 106 abdominal vascular injuries, all 41 patients arriving in shock following gunshot wounds were candidates for rapid transfer to the operating theatre for surgical bleeding control [62]. A similar observation in a study of 271 patients undergoing immediate laparotomy for gunshot wounds indicates that these wounds combined with signs of severe hypovolaemic shock specifically require early surgical bleeding control. This observation is true to a lesser extent for abdominal stab wounds [63]. Data on injuries caused by penetrating metal fragments from explosives or gunshot wounds in the Vietnam War confirm the need for early surgical control when patients present in shock [64]. In blunt trauma, the mechanism of injury can to a certain extent determine whether the patient in haemorrhagic shock will be a candidate for surgical bleeding control. Only a few studies address the relationship between the mechanism of injury and the risk of bleeding, however, and none of these publications is a randomised prospective trial of high evidence [65]. We have found no objective data describing the relationship between the risk of bleeding and the mechanism of injury resulting in skeletal fractures in general or of long-bone fractures in particular.

Traffic accidents are the leading cause of pelvic injury. Motor vehicle crashes cause approximately 60% of pelvic fractures followed by falls from great height (23%). Most of the remainder result from motorbike collisions and vehicle-pedestrian accidents [66,67]. There is a correlation between 'unstable' pelvic fractures and intra-abdominal injuries [66,68]. An association between major pelvic fractures and severe head injuries, concomitant thoracic, abdominal, urological and skeletal injuries is also well described [66]. High-energy injuries produce greater damage to both the pelvis and organs. Patients with high-energy injuries require more transfusion units, and more than 75% have associated head, thorax, abdominal or genitourinary injuries [69]. It is well documented that 'unstable' pelvic fractures are associated with massive hemorrhage [68,70], and hemorrhage is the leading cause of death in patients with major pelvic fractures. Vertical shear pelvic ring fractures with caudal displacement of the hemipelvis may disrupt the pelvic floor and pelvic vasculature far more than standard vertical shear injuries. Inferior displacement of the hemipelvis using X-ray imaging should, therefore, alert the surgeon to the possible presence of severe arterial injuries [71].

#### Further investigation

##### Recommendation 6

We recommend that patients presenting with haemorrhagic shock and an unidentified source of bleeding undergo immediate further investigation. (Grade 1B)

###### Rationale

A patient in haemorrhagic shock with an unidentified source of bleeding should undergo immediate further assessment of chest, abdominal cavity and pelvic ring, which represent the major sources of acute blood loss in trauma. Aside from a clinical examination, X-rays of chest and pelvis in conjunction with ultrasonography [72] or occasionally diagnostic peritoneal lavage (DPL) [73] are recommended diagnostic modalities during the primary survey [57,74,75]. In selected centres, readily available computed tomography (CT) scanners [76] may replace conventional radiographic imaging techniques during the primary survey. In their systematic literature review, Jorgensen and colleagues found no evidence that pre-hospital ultrasound of the abdomen or chest improves the treatment of trauma patients [77].

#### Imaging

##### Recommendation 7

We recommend early imaging (ultrasonography or CT) for the detection of free fluid in patients with suspected torso trauma. (Grade 1B)

#### Intervention

##### Recommendation 8

We recommend that patients with significant free intra-abdominal fluid and haemodynamic instability undergo urgent intervention. (Grade 1A)

#### Further assessment

##### Recommendation 9

We recommend further assessment using CT for haemodynamically stable patients. (Grade 1B)

###### Rationale

Blunt abdominal trauma represents a major diagnostic challenge and an important source of internal bleeding. Ultrasonography has been established as a rapid and non-invasive diagnostic approach for detection of intra-abdominal free fluid in the emergency room [78-80]. Large prospective observational studies determined a high specificity and accuracy but low sensitivity of initial ultrasonographic examination for detecting intra-abdominal injuries in adults and children [81-87]. Liu and colleagues [88] found a high sensitivity, specificity and accuracy of initial ultrasound examination for the detection of haemoperitoneum. Ultrasonography has a high specificity but a low sensitivity for detecting free intra-peritoneal fluid in penetrating torso trauma [89] and in blunt abdominal trauma in children [90]. A positive ultrasound suggests haemoperitoneum, but a negative initial abdominal ultrasound should direct further diagnostic investigations. Although CT scan and DPL were shown to be more sensitive than sonography for detection of haemoperitoneum, these diagnostic modalities are more time-consuming (CT and DPL) and invasive (DPL) [88].

The role of CT-scanning in acute trauma patients is well documented [91-98], and in recent years imaging for trauma patients has migrated towards multi-slice computed tomography (MSCT). The integration of modern MSCT scanners in the emergency room area allows the immediate assessment of trauma victims following admission [93,94]. Using modern MSCT scanners, total whole-body scanning time may be reduced to less than 30 seconds. In a retrospective study comparing 370 patients in two groups, Weninger and colleagues [94] showed that faster diagnosis using MSCT led to shorter emergency room and operating room time and shorter intensive care unit (ICU) stays [94]. Huber-Wagner *et al*. [76] also showed the benefit of integration of the whole-body CT into early trauma care. CT diagnosis significantly increases the probability of survival in patients with polytrauma. Whole-body CT as a standard diagnostic tool during the earliest resuscitation phase for polytraumatised patients provides the added benefit of identifying head and chest injuries and other bleeding sources in multiply injured patients.

Some authors have shown the benefit of contrast medium-enhanced CT scanning. Anderson *et al*. [99,100] found high accuracy in the evaluation of splenic injuries resulting from trauma after administration of IV contrast material. Delayed-phase CT may be used to detect active bleeding in solid organs. Fang *et al*. [101] demonstrated that the pooling of contrast material within the peritoneal cavity in blunt liver injuries indicates active and massive bleeding. Patients with this finding showed rapid deterioration of haemodynamic status, and most of them required emergent surgery. Intra-parenchymal pooling of contrast material with an unruptured liver capsule often indicates a self-limited hemorrhage, and these patients respond well to non-operative treatment. Tan and colleagues [102] found that patients with hollow viscus and mesenteric injuries following blunt abdominal trauma exhibited an abnormal preoperative CT scan. Wu *et al*. [103] showed the accuracy of CT in identifying severe, life-threatening mesenteric hemorrhage and blunt bowel injuries.

Compared to MSCT, all traditional techniques for diagnostic and imaging evaluation are associated with some limitations. The diagnostic accuracy, safety and effectiveness of immediate MSCT are dependent on sophisticated pre-hospital treatment by trained and experienced emergency personnel and short transportation times [104,105]. If an MSCT is not available in the emergency room, the realisation of CT scanning implies transportation of the patient to the CT room; therefore, the clinician must evaluate the implications and potential risks and benefits of the procedure. During transport, all vital signs should be closely monitored and resuscitation measures continued. For those patients in whom haemodynamic stability is questionable, imaging techniques, such as ultrasound and chest and pelvic radiography, may be useful. Peritoneal lavage is rarely indicated if ultrasound or CT is available [106]. Transfer times to and from all forms of diagnostic imaging need to be considered carefully in any patient who is haemodynamically unstable. In addition to the initial clinical assessment, near-patient testing results, including full blood count, haematocrit (Hct), blood gases and lactate, should be readily available under ideal circumstances.

The hypotensive patient (systolic blood pressure below 90 mmHg) presenting free intra-abdominal fluid according to ultrasonography or CT is a potential candidate for early surgery if he or she cannot be stabilised by initiated fluid resuscitation [107-109]. A retrospective study by Rozycki and colleagues [110] of 1,540 patients (1,227 with blunt, 313 with penetrating trauma) assessed with ultrasound as an early diagnostic tool showed that the ultrasound examination had a sensitivity and specificity close to 100% when patients were hypotensive.

A number of patients who present with free intra-abdominal fluid according to ultrasound can safely undergo further investigation with MSCT. Under normal circumstances, adult patients need to be haemodynamically stable when MSCT is performed outside of the emergency room [110]. Haemodynamically stable patients with a high risk mechanism of injury, such as high-energy trauma or even low-energy injuries in the older population, should be scanned after ultrasound for additional injuries using MSCT. As CT scanners are integrated in resuscitation units, whole-body CT diagnosis may replace ultrasound as a diagnostic method.

#### Haematocrit

##### Recommendation 10

We do not recommend the use of single Hct measurements as an isolated laboratory marker for bleeding. (Grade 1B)

###### Rationale

Hct assays are part of the basic diagnostic work-up for trauma patients. The diagnostic value of the Hct for detecting trauma patients with severe injury and occult bleeding sources has been a topic of debate in the past decade [111-113]. A major limit of the Hct's diagnostic value is the confounding influence of resuscitative measures on the Hct due to administration of intravenous fluids and red cell concentrates [114-116]. In addition, initial Hct does not accurately reflect blood loss because patients bleed whole blood and compensatory mechanisms that move fluids from interstitial space require time and are not reflected in initial Hct measurements. A retrospective study of 524 trauma patients determined a low sensitivity (0.5) of the initial Hct on admission for detecting those patients with traumatic hemorrhage requiring surgical intervention [113]. The concept of the low sensitivity of initial Hct for the detection of severe bleeding has recently been challenged. In a retrospective study of 196 trauma patients, Ryan *et al*. [117] found that Hct at admission closely correlates with haemorrhagic shock. However, this study included severe cases requiring emergency surgery only (most with penetrating injuries), and may not be applicable to the general trauma patient population. Two prospective observational diagnostic studies determined the sensitivity of serial Hct measurements for detecting patients with severe injury [111,112]. Decreasing serial Hct measurements may reflect continued bleeding; however, the patient with significant bleeding may maintain his or her serial Hct.

#### Serum lactate and base deficit

##### Recommendation 11

We recommend either serum lactate or base deficit measurements as sensitive tests to estimate and monitor the extent of bleeding and shock. (Grade 1B)

###### Rationale

Serum lactate has been used as a diagnostic parameter and prognostic marker of haemorrhagic shock since the 1960s [118]. The amount of lactate produced by anaerobic glycolysis is an indirect marker of oxygen debt, tissue hypoperfusion and the severity of haemorrhagic shock [119-122]. Similarly, base deficit values derived from arterial blood gas analysis provide an indirect estimation of global tissue acidosis due to impaired perfusion [119,121]. Vincent and colleagues [123] showed the value of serial lactate measurements for predicting survival in a prospective study in patients with circulatory shock. This study showed that changes in lactate concentrations provide an early and objective evaluation of a patient's response to therapy and suggested that repeated lactate determinations represent a reliable prognostic index for patients with circulatory shock [123]. Abramson and colleagues [124] performed a prospective observational study in patients with multiple trauma to evaluate the correlation between lactate clearance and survival. All patients in whom lactate levels returned to the normal range (≤2 mmol/l) within 24 h survived. Survival decreased to 77.8% if normalisation occurred within 48 h and to 13.6% in those patients in whom lactate levels were elevated above 2 mmol/l for more than 48 h [124]. These findings were confirmed in a study by Manikis and colleagues [125], who showed that the initial lactate levels were higher in non-survivors after major trauma, and that the prolonged time for normalisation of lactate levels of more than 24 h was associated with the development of post-traumatic organ failure [125]. The usefulness of lactate determination in trauma patients is well established; however, the reliability of this measure may be lower when traumatic injury is associated with alcohol consumption, because alcohol itself can increase the level of lactate in the blood. In alcohol associated-trauma, therefore, base deficit may be a better predictor of prognosis than lactate [126].

Similar to the predictive value of lactate levels, the initial base deficit, obtained either from arterial or peripheral venous blood [127] has been established as a potent independent predictor of mortality in patients with traumatic-hemorrhagic shock [126]. Davis and colleagues [128] stratified the extent of base deficit into three categories: mild (-3 to -5 mEq/l), moderate (-6 to -9 mEq/l) and severe (<-10 mEq/l), and established a significant correlation between the admission base deficit, transfusion requirements within the first 24 h and the risk of post-traumatic organ failure or death [128]. The same group of authors showed that the base deficit is a better prognostic marker of death than the pH in arterial blood gas analyses [129]. Furthermore, the base deficit was shown to represent a highly sensitive marker for the extent of post-traumatic shock and mortality, both in adult and paediatric patients [130,131].

In contrast to the data on lactate levels in haemorrhagic shock, reliable large-scale prospective studies on the correlation between base deficit and outcome are still lacking. Although both the base deficit and serum lactate levels are well correlated with shock and resuscitation, these two parameters do not strictly correlate with each other in severely injured patients [132]. Therefore, the independent assessment of both parameters is recommended for the evaluation of shock in trauma patients [119,121,132].

#### Coagulation monitoring

##### Recommendation 12

We recommend that routine practice to detect post-traumatic coagulopathy include the early, repeated and combined measurement of prothrombin time (PT), activated partial thromboplastin time (APTT), fibrinogen and platelets. (Grade 1C)

We recommend that viscoelastic methods also be performed to assist in characterising the coagulopathy and in guiding haemostatic therapy. (Grade 1C)

###### Rationale

Standard coagulation monitoring comprises the early and repeated determination of PT, APTT, platelet counts and fibrinogen. Increasing emphasis focuses on the importance of fibrinogen and platelet measurements. It is often assumed that the conventional coagulation screens (international normalised ratio (INR) and APTT) monitor coagulation; however, these tests monitor only the initiation phase of blood coagulation, and represent only the first 4% of thrombin production [133]. It is, therefore, possible that the conventional coagulation screen appears normal, while the overall state of blood coagulation is abnormal [134-139]. In addition, the delay in detection of traumatic coagulopathy can influence outcome, and the turn-around time of thromboelastometry has been shown to be significantly shorter compared to conventional laboratory testing, with a time savings of about 30 to 60 minutes [136,140,141]. Viscoelastic testing may also be useful in the detection of coagulation abnormalities associated with the use of direct thrombin inhibitors, such as dabigatran, argatroban, bivalirudin or hirudin. Furthermore, (early) variables of clot firmness assessed by viscoelastic testing have been shown to be good predictors for the need for massive transfusion, the incidence of thrombotic/thromboembolic events and for mortality in surgical and trauma patients [136,142-151]. Therefore, complete and rapid monitoring of blood coagulation and fibrinolysis using viscoelastic methods may facilitate a more accurate targeting of therapy.

Tools, such as thromboelastometry and portable coagulometers, have been developed to detect coagulopathy in the emergency room or at the bedside, improving the availability of real-time data to guide patient management. Portable coagulometers that provide INR or APTT seem to provide acceptable accuracy for point-of-care INR testing in the emergency department compared with laboratory-based methods [152,153], but are limited by the usefulness of the parameters measured.

The number of publications describing the use of viscoelastic methodology is rapidly increasing; however, the methods employed by different investigators differ significantly, highlighting a need for standardisation of the technique [154,155]. Case series using viscoelastic testing to assess trauma patients have been published. One study applied rotation thrombelastography to 23 patients, but without a comparative standard [134]. Another study found a poor correlation between rotation thrombelastography and conventional coagulation parameters [14]. Johanssen *et al*. [135] implemented a haemostatic resuscitation regime (early platelets and fresh frozen plasma (FFP)) guided using thrombelastography in a before-and-after study (n = 832), which showed improved outcomes. In a retrospective study of cardiovascular surgery patients (n = 3,865), the combined use of thromboelastometry and portable coagulometry resulted in a reduction in blood product transfusion and thromboembolic events, but did not influence mortality [156]. Rapid thrombelastography is a new variant of viscoelastic testing in which coagulation is initiated by the addition of kaolin and tissue factor that appears to reduce the measurement time compared with conventional thrombelastography [157]. Despite the wide-spread use of viscoelastic methods, some limitations must be kept in mind. Larsen *et al*. found that thrombelastography was unable to distinguish coagulopathies caused by dilution from thrombocytopenia, whereas thromboelstometry was indeed capable of distinguishing these two different types of coagulopathy and suggesting the correct treatment [158]. The use of thrombelastography may thus lead to unnecessary transfusion with platelets, whereas the application of thromboelastometry may result in goal-directed fibrinogen substitution. Although rapidly increasing, at present controversy remains regarding the utility of viscoelastic methods for the detection of post-traumatic coagulopathy. One limitation of viscoelastic tests is the lack of sensitivity to detect and monitor platelet dysfunction due to antiplatelet drugs. If platelet dysfunction is expected, point-of-care platelet function tests, for example, whole blood impedance aggregometry, should be used in addition to viscoelastic tests [159,160]. More research is required in this area, and in the meantime physicians should use their own judgement when developing local policies.

It is theoretically possible that the pattern of change in measures of coagulation, such as D-dimers, may help to identify patients with ongoing bleeding. However, a single publication showed that the positive predictive value of D-dimers is only 1.8% in the postoperative and/or posttraumatic setting [161]; therefore, traditional methods of detection for ongoing bleeding, such as serial clinical evaluation of radiology (ultrasound, CT or angiography) should be used.

### III. Tissue oxygenation, fluid and hypothermia

#### Tissue oxygenation

##### Recommendation 13

We recommend a target systolic blood pressure of 80 to 90 mmHg until major bleeding has been stopped in the initial phase following trauma without brain injury. (Grade 1C)

We recommend that a mean arterial pressure ≥80 mmHg be maintained in patients with combined haemorrhagic shock and severe TBI (GCS ≤8). (Grade 1C)

###### Rationale

In order to maintain tissue oxygenation, traditional treatment of trauma patients used early and aggressive fluid administration to restore blood volume. This approach may, however, increase the hydrostatic pressure on the wound, cause dislodgement of blood clots, a dilution of coagulation factors and undesirable cooling of the patient. The concept of low volume fluid resuscitation, so-called "permissive hypotension", avoids the adverse effects of early aggressive resuscitation while maintaining a level of tissue perfusion that, although lower than normal, is adequate for short periods [162]. Its general effectiveness remains to be confirmed in randomised clinical trials; however, two studies published in the 1990s demonstrated increased survival when a low and delayed volume fluid resuscitation concept was used in penetrating [163] or penetrating and blunt [164] trauma. However, in contrast to these studies, no significant differences in survival were found in two further trials in patients with either penetrating and blunt trauma [165] or blunt trauma alone [166].

Ten years ago a Cochrane systematic review concluded that there is no evidence from randomised clinical trials for or against early or larger amounts of intravenous fluids to treat uncontrolled hemorrhage [167]. However, more recent retrospective analyses demonstrated that aggressive resuscitation techniques, often initiated in the pre-hospital setting, may be detrimental for trauma patients [5,17,168,169]. One of these studies showed that this strategy increased the likelihood that patients with severe extremity injuries developed secondary abdominal compartment syndrome (ACS) [168]. In that study, early large-volume crystalloid administration was the greatest predictor of secondary ACS. Moreover, another retrospective analysis of the German Trauma Registry database, including 17,200 multiply-injured patients, showed that the incidence of coagulopathy increased with increasing volume of IV fluids administered pre-clinically [5]. Coagulopathy was observed in >40% of patients with >2,000 ml, in >50% with >3,000 ml and in >70% with >4,000 ml administered. Using the same trauma registry, a retrospective matched pairs analysis (n = 1,896) demonstrated that multiply-injured trauma patients with an Injury Severity Score (ISS) ≥16 points and a systolic blood pressure ≥60 mmHg at the accident site who received pre-hospital low-volume resuscitation (0 to 1,500 ml) had a higher survival rate than patients in whom a pre-hospital high-volume strategy (≥1,501 ml) was used [17]. These results are supported by another retrospective analysis of patients from the US National Trauma Data Bank [169]. In this study, the authors analysed 776,734 patients, of whom about 50% received pre-hospital IV fluid and 50% did not. The group of patients receiving preoperative IV fluids were significantly more likely to die (OR 1.11, 95% CI 1.05 to 1.17), an association which was especially marked in patients with penetrating mechanisms of injury (OR 1.25, 95% CI 1.08 to 1.45), hypotension (OR 1.44, 95% CI 1.29 to 1.59), severe head injury (OR 1.34, 95% CI 1.17 to 1.54) and patients undergoing immediate surgery (OR 1.35, 95% CI 1.22 to 1.50). The authors concluded that the routine use of pre-hospital IV fluid for all trauma patients should be discouraged.

Evidence for the restricted initial administration of intra-hospital fluid is more clear. A recently published prospective randomised trial analysing the consequences of a hypotensive resuscitation strategy in trauma patients with hemorrhagic shock demonstrated a benefit for the initial intra-hospital hypotensive resuscitation strategy [170]. In this study, with nearly all of the 90 patients suffering from penetrating trauma, patients who had at least one documented in-hospital systolic blood pressure ≤90 mmHg were randomised to a group whose target minimum mean arterial pressure was 50 mmHg or 65 mmHg. One major drawback to this study was that no statistically significant differences between the actual mean arterial pressure was observed between the two groups for the duration of the study (64.4 mmHg vs. 68.5 mmHg, *P *= 0.15). Although the authors could not demonstrate a survival difference for the two treatment strategies at Day 30, 24 h postoperative death and coagulopathy were increased in the group with the higher target minimum pressure. The patients in this group received not only more IV fluids overall, but also more blood product transfusions.

In spite of these recently published data that include patients with TBI, the low volume approach in hypotensive patients is contraindicated in TBI and spinal injuries, because an adequate perfusion pressure is crucial to ensure tissue oxygenation of the injured central nervous system [171]. In addition, the concept of permissive hypotension should be carefully considered in the elderly patient, and may be contraindicated if the patient suffers from chronic arterial hypertension [172].

#### Fluid therapy

##### Recommendation 14

We recommend that fluid therapy be initiated in the hypotensive bleeding trauma patient. (Grade 1A)

We recommend that crystalloids be applied initially to treat the hypotensive bleeding trauma patient. (Grade 1B)

We recommend that hypotonic solutions, such as Ringer's lactate, be avoided in patients with severe head trauma. (Grade 1C)

If colloids are administered, we recommend use within the prescribed limits for each solution. (Grade 1B)

We suggest that hypertonic solutions during initial treatment be used, but demonstrate no advantage compared to crystalloids or colloids in blunt trauma and TBI. (Grade 2B)

We suggest the use of hypertonic solutions in hemodynamically unstable patients with penetrating torso trauma. (Grade 2C)

###### Rationale

Although fluid resuscitation is the first step to restore tissue perfusion in severe haemorrhagic shock, it is still unclear whether colloids or crystalloids, and more specifically, which colloid or which crystalloid, should be used in the initial treatment of the bleeding trauma patient. The most recent Cochrane meta-analysis on the type of fluid, colloids or crystalloids, could not demonstrate that colloids reduce the risk of death compared to resuscitation with crystalloids [173]. The authors compared albumin with plasma protein fraction, performing an analysis of 23 trials that included a total of 7,754 patients. Hydroxyethyl starch (HES) was evaluated in an analysis of 17 trials that included a total of 1,172 patients, modified gelatine was assessed in 11 trials that included a total of 506 patients, and 9 trials that included a total of 834 patients examined the effectiveness of dextran. The authors concluded that the use of colloids is only justified in the context of RCTs, since they could not show any beneficial effect of colloids, which are also more expensive than crystalloids. Therefore, the initial administration of crystalloids to treat the hypotensive bleeding trauma patient seems to be justified. Moreover, it was shown that large volume crystalloid administration is not independently associated with multiple organ failure [174]. In contrast, if high ratios of FFP:RBC (red blood cells) cannot be administered to trauma patients, resuscitation with at least 1 l crystalloid per unit RBC seems to be associated with reduced overall mortality [175]. If crystalloids are used, hypotonic solutions, such as Ringer's lactate, should be avoided in patients with TBI in order to minimize a fluid shift into the damaged cerebral tissue. In addition, the use of solutions with the potential to restore pH may be advantageous, since a recent study demonstrated that Ringer's acetate solution more rapidly ameliorated splanchnic dysoxia, as evidenced by gastric tonometry, than Ringer's lactate [176]. Whether an advantage exists for certain isotonic crystalloids associated with reduced morbidity or mortality remains to be evaluated.

So far it is not clear whether, and if so, which colloids should be used after initial infusion of crystalloids. Bunn *et al*. published a Cochrane meta-analysis with the aim of comparing the effects of different colloid solutions in patients thought to need volume replacement [177]. From this review, there is no evidence that one colloid solution is more effective or safer than any other, although the confidence intervals were wide and do not exclude clinically significant differences between colloids. In contrast, another recent meta-analysis, which included 69 clinical studies with a total of 10,382 patients published since 2002, showed that acute kidney injury and impaired coagulation associated with different HES solutions as possible side effects [178]. However, this analysis was largely influenced by data from the so-called VISEP trial in septic patients [179]. In this trial an older hypertonic HES solution (200/0.5) was used and frequently administered in excess of the maximal permissible dose. Nevertheless, another study in septic patients showed similar negative results [180]. So far, only one recently published small RCT described a benefit for a HES solution. HES (130/0.4) provided significantly better lactate clearance and less renal injury than saline in 67 penetrating trauma patients [181]. Because only 42 blunt trauma patients were included in the study, no differences in these parameters could be observed using the different solutions. Therefore, if colloids are administered, dosing should be within the prescribed limits and, if HES is employed, a modern HES solution should be used.

Promising results have been obtained using hypertonic solutions. In 2008, a double-blind RCT in 209 patients with blunt traumatic injuries analysed the effect of treatment with 250 ml 7.5% hypertonic saline and 6% dextran 70 compared to lactated Ringer's solution on organ failure [182]. The intent-to-treat analysis demonstrated no significant difference in organ failure and in acute respiratory distress syndrome (ARDS)-free survival. However, there was improved ARDS-free survival in the subset (19% of the population) requiring 10 U or more of packed RBC [182]. Another study comparing hypertonic saline dextran with normal saline for resuscitation in hypotension from penetrating torso injuries showed improved survival in the hypertonic saline dextran group when surgery was required [183]. A clinical trial with brain injury patients found that hypertonic saline reduced intracranial pressure more effectively than dextran solutions with 20% mannitol when compared in equimolar dosing [184]. However, Cooper *et al*. found almost no difference in neurological function six months after TBI in patients who had received pre-hospital hypertonic saline resuscitation compared to conventional fluid [185]. The validity of these results was supported by the meta-analysis of Perel and Roberts, which did not demonstrate beneficial effects of hypertonic solutions [173]. The eight trials with 1,283 randomised participants compared dextran in hypertonic crystalloid with isotonic crystalloid and demonstrated a pooled RR of 1.24 (95% CI 0.94 to 1.65). Moreover, two recently published large prospective randomised multi-centre studies by Bulger and co-workers [186,187] that were not included in this meta-analysis analysed the effect of out-of-hospital administration of hypertonic fluids on neurologic outcome following severe TBI and survival after traumatic hypovolaemic shock. These studies were not able to demonstrate any advantage compared to normal 0.9% saline among the 2,184 patients included. In conclusion, the evidence suggests that hypertonic saline solutions are safe, but will neither improve survival nor improve neurological outcome after TBI.

#### Vasopressors and inotropic agents

##### Recommendation 15

We suggest administration of vasopressors to maintain target arterial pressure in the absence of a response to fluid therapy. (Grade 2C)

We suggest infusion of an inotropic agent in the presence of myocardial dysfunction. (Grade 2C)

###### Rationale

The first step in shock resuscitation is to rapidly restore mean arterial pressure and systemic blood flow to prevent regional hypoperfusion and tissue hypoxia. Fluid resuscitation is the first strategy applied to restore mean arterial pressure in hemorrhagic shock. However, vasopressor agents may also be transiently required to sustain life and maintain tissue perfusion in the presence of life-threatening hypotension, even when fluid expansion is in progress and hypovolaemia has not yet been corrected.

Norepinephrine (NE) is often used to restore arterial pressure in septic and haemorrhagic shock. It is now recommended as the agent of choice for this purpose during septic shock [188]. NE is a sympathomimetic agent with predominantly vasoconstrictive effects. Arterial α-adrenergic stimulation increases arterial resistance and may increase cardiac afterload, and NE exerts both arterial and venous α-adrenergic stimulation [189]. Indeed, in addition to its arterial vasoconstrictor effect, NE induces venoconstriction at the level of the splanchnic circulation in particular, which increases the pressure in capacitance vessels and actively shifts splanchnic blood volume to the systemic circulation [190]. This venous adrenergic stimulation may recruit some blood from the venous unstressed volume, that is, the blood volume filling the blood vessels, without generating an intravascular pressure. Moreover, stimulation of β_2_-adrenergic receptors decreases venous resistance and increases venous return [190].

Animal studies using models of uncontrolled hemorrhage have suggested that NE infusion reduces the amount of fluid resuscitation required to achieve a given arterial pressure target, is associated with lower blood loss and significantly improves survival [191]. However, the effects of NE have not been rigorously investigated in humans with haemorrhagic shock. An interim analysis performed during an ongoing multi-centre prospective cohort study suggested that the early use of vasopressors for haemodynamic support after haemorrhagic shock may be deleterious compared to aggressive volume resuscitation and should be used cautiously [192]. This study has several limitations, however. First, this was a secondary analysis of a prospective cohort study and was not designed to answer the specific hypothesis tested and, second, the group receiving vasopressors had a higher rate of thoracotomy. Thus, a prospective study to define the effect of vasopressors in haemorrhagic shock is clearly needed. Vasopressors may be useful if used transiently to sustain arterial pressure and maintain tissue perfusion in face of a life-threatening hypotension. If used, it is essential to respect the recommended objectives for arterial pressure (systolic arterial pressure 80 to 90 mmHg).

Because vasopressors may increase cardiac afterload if the infusion rate is excessive or left ventricular function is already impaired, an assessment of cardiac function during the initial ultrasound examination is essential. Cardiac dysfunction could be altered in the trauma patient following cardiac contusion, pericardial effusion or secondary to brain injury with intracranial hypertension. The presence of myocardial dysfunction requires treatment with an inotropic agent, such as dobutamine or epinephrine. In the absence of an evaluation of cardiac function or cardiac output monitoring, as is often the case in the early phase of haemorrhagic shock management, cardiac dysfunction must be suspected in the presence of a poor response to fluid expansion and NE.

#### Temperature management

##### Recommendation 16

We recommend early application of measures to reduce heat loss and warm the hypothermic patient in order to achieve and maintain normothermia. (Grade 1C)

**We suggest that hypothermia at 33 to 35**º**C for ≥48 h be applied in patients with TBI once bleeding from other sources has been controlled. (Grade 2C)**

###### Rationale

Hypothermia, defined as a core body temperature <35ºC, is associated with acidosis, hypotension and coagulopathy in severely injured patients. In a retrospective study with 122 patients, hypothermia was an ominous clinical sign, accompanied by high mortality and blood loss [193]. The profound clinical effects of hypothermia ultimately lead to higher morbidity and mortality, and hypothermic patients require more blood products [194].

Hypothermia is associated with an increased risk of severe bleeding, and hypothermia in trauma patients represents an independent risk factor for bleeding and death [195]. The effects of hypothermia include altered platelet function, impaired coagulation factor function (a 1ºC drop in temperature is associated with a 10% drop in function), enzyme inhibition and fibrinolysis [196,197]. Body temperatures below 34ºC compromise blood coagulation, but this has only been observed when coagulation tests (PT and APTT) are carried out at the low temperatures seen in patients with hypothermia, and not when assessed at 37ºC as is routine practice for such tests. Steps to prevent hypothermia and the risk of hypothermia-induced coagulopathy include removing wet clothing, covering the patient to avoid additional heat loss, increasing the ambient temperature, forced air warming, warm fluid therapy and, in extreme cases, extracorporeal re-warming devices [198,199].

Whereas hypothermia should be avoided in patients without TBI, contradictory results have been observed in meta-analyses that examine mortality and neurological outcomes associated with mild hypothermia in TBI, possibly due to the different exclusion and inclusion criteria for the studies used for the analysis [200-202]. The speed of induction and duration of hypothermia may be important factors that influence the benefit associated with this treatment. It has been shown that five days of long-term cooling is more efficacious than two days of short-term cooling when mild hypothermia is used to control refractory intracranial hypertension in adults with severe TBI [203]. Obviously, the time span of hypothermia is crucial, because a recent prospective RCT in 225 children with severe TBI showed that hypothermic therapy initiated within 8 h after injury and continued for 24 h did not improve the neurological outcome and may increase mortality [204]. Furthermore, the mode of cerebral hypothermia induction may influence its effectiveness. In a RCT comparing non-invasive selective brain cooling (33 to 35°C) in 66 patients with severe TBI and mild systemic hypothermia (rectal temperature 33 to 35°C) and a control group not exposed to hypothermia, natural rewarming began after three days. Mean intracranial pressure (ICP) 24, 48 or 72 h after injury was significantly lower in the selective brain cooling group than in the control group [205]. In another study, the difference in the intracranial pressure using two different levels of hypothermia was examined. However, this observational study failed to demonstrate differences in ICP reduction using either 35°C or 33°C hypothermia [206].

The most recent meta-analysis divided the 12 RCTs analysing the effect of mild hypothermia compared to standard treatment for TBI in 1,327 patients into 2 subgroups based on cooling strategy: short term (≤48 h) and long-term or goal-directed (>48 h and/or continued until normalisation of ICP) [207]. Although the authors demonstrated a lower mortality (RR 0.73, 95% CI 0.62 to 0.85) and more positive neurologic outcomes (RR 1.52, 95% CI 1.28 to 1.80) for all 12 studies in favour of the hypothermia-treated patients, these beneficial effects could neither be shown with respect to mortality (RR 0.98, 95% CI 0.75 to 1.30) nor neurologic outcome (RR 1.31, 95% CI 0.94 to 1.83) if only the short-term cooling studies were analysed. In contrast, among the eight studies of long-term or goal-directed cooling, mortality was reduced (RR 0.62, 95% CI 0.51 to 0.76) and good neurologic outcome was more common (RR 1.68, 95% CI 1.44 to 1.96). These results are in line with a meta-analysis performed two years earlier [208]. Unfortunately, these results were not confirmed by the National Acute Brain Injury Study: Hypothermia II (NABIS: H II), which was a RCT of 232 patients with severe brain injury who were enrolled within 2.5 h of injury and either randomly assigned to hypothermia (35°C followed by 33°C for 48 h and then gradually rewarmed) or treated at normothermia [209]. Due to secondary exclusion criteria, only 52 patients remained in the hypothermia group and only 45 in the normothermia group, which was also one reason that the trial was stopped for futility after 3.5 years. Neither mortality nor the neurological outcome demonstrated a benefit for hypothermia as a primary neuroprotective strategy in patients with severe TBI.

In conclusion, prolonged hypothermia may be considered in patients with isolated head trauma after hemorrhage has been arrested. If mild hypothermia is applied in TBI, cooling should take place within the first 3 h following injury, preferably using selective brain cooling by cooling the head and neck, be maintained at least for >48 h, rewarming should last 24 h and the cerebral perfusion pressure should be maintained at >50 mmHg (systolic blood pressure ≥70 mmHg). Patients most likely to benefit from hypothermia are those with a Glasgow Coma Score (GCS) at admission between 4 and 7 [202]. Possible side effects are hypotension, hypovolaemia, electrolyte disorders, insulin resistance and reduced insulin secretion and increased risk of infection [202]. Nevertheless, a recent case control study did not reveal any evidence that a 48-h hypothermic period increases the risk of infection in patients after TBI treated with selective gut decontamination [210]. Further studies are warranted to investigate the postulated benefit of hypothermia in TBI taking these important factors into account.

#### Erythrocytes

##### Recommendation 17

We recommend a target haemoglobin (Hb) of 7 to 9 g/dl. (Grade 1C)

###### Rationale

Oxygen delivery to the tissues is the product of blood flow and arterial oxygen content, which is directly related to the Hb concentration. A decrease in Hb may, therefore, be expected to result in tissue hypoxia. However, physiologic responses to acute normovolaemic anaemia, including macro- and microcirculatory changes in blood flow, can compensate for the decrease in Hb concentration.

No prospective RCT has compared restrictive and liberal transfusion regimens in trauma, but 203 trauma patients from the Transfusion Requirements in Critical Care trial [211] were re-analysed [212]. A restrictive transfusion regimen (Hb transfusion trigger <7.0 g/dl) resulted in fewer transfusions as compared with the liberal transfusion regimen (Hb transfusion trigger <10 g/dl) and appeared to be safe. However, no statistically significant benefit in terms of multiple organ failure or post-traumatic infections was observed. It should be emphasised that this study was neither designed nor powered to answer these questions with precision. In addition, it cannot be ruled out that the number of RBC units transfused merely reflects the severity of injury. Nevertheless, RBC transfusions have been shown in multiple studies to be associated with increased mortality [213-217], lung injury [217-219], increased infection rates [220,221] and renal failure in trauma victims [216]. This ill effect may be particularly important with RBCs stored for more than 14 days [216].

Despite the lack of high-level scientific evidence for a specific Hb transfusion trigger in patients with TBI, these patients are currently transfused in many centres to achieve a Hb of approximately 10 g/dl [222]. This might be justified by the finding that increasing the Hb from 8.7 to 10.2 g/dl improved local cerebral oxygenation in 75% of patients [223]. In another preliminary study in patients with TBI, one to two RBC transfusions at a Hb of approximately 9 g/dl transiently (three to six hours) increased cerebral oxygenation, again in approximately 75% of patients [224,225]. A storage time of more than 19 days precluded this effect [224]. In another recent study, cerebral tissue oxygenation, on average, did not increase due to an increase in Hb from 8.2 to 10.1 g/dl [226]. Nevertheless, the authors came to the conclusion, based on multivariate statistical models, that the changes in cerebral oxygenation correlated significantly with Hb concentration [226]. This conclusion, however, was questioned in the accompanying editorial [227]. In an initial outcome study the lowest Hct was correlated with adverse neurological outcome and RBC transfusions were also found to be an independent factor predicting adverse neurological outcome [228]. Interestingly, the number of days with a Hct below 30% was found to be correlated with an improved neurological outcome [228]. In an outcome study of 1,150 patients with TBI, RBC transfusions were found to be associated with a two-fold increased mortality and a three-fold increased complication rate [229]. A recent retrospective observational analysis of 139 TBI patients suggests that increasing Hct above 28% during the initial unstable operating room phase following severe TBI is not associated with improved outcome as determined by the extended Glasgow outcome scale after six months [230]. In another retrospective study of 234 patients with severe TBI, anaemia (defined as a Hb level <10 g/dl) in the emergency department or ICU is not a risk factor for poor outcome [231]. Therefore, patients with severe TBI should not be managed with an Hb transfusion threshold different than that of other critically ill patients.

Erythrocytes contribute to haemostasis by influencing the biochemical and functional responsiveness of activated platelets via the rheological effect on platelet margination and by supporting thrombin generation [232]; however, the optimal Hct or Hb concentration required to sustain haemostasis in massively bleeding patients is unclear. Further investigations into the role of the Hb concentration on haemostasis in massively transfused patients are, therefore, warranted.

The effects of the Hct on blood coagulation have not been fully elucidated [233]. An acute reduction of the Hct results in an increase in the bleeding time [234,235], with restoration upon re-transfusion [234]. This may relate to the presence of the enzyme elastase on the surface of RBC membranes, which may activate coagulation factor IX [236,237]. However, a moderate reduction of the Hct does not increase blood loss from a standard spleen injury [235], and an isolated *in vitro *reduction of the Hct did not compromise blood coagulation as assessed by thromboelastometry [238].

### IV. Rapid control of bleeding

#### Early abdominal bleeding control

##### Recommendation 18

We recommend that early bleeding control of the abdomen be achieved using packing, direct surgical bleeding control and the use of local haemostatic procedures. In the exsanguinating patient, aortic cross-clamping may be employed as an adjunct. (Grade 1C)

Abdominal resuscitative packing is an early part of the post-traumatic laparotomy to identify major injuries and sources of hemorrhage [239,240]. If bleeding cannot be controlled using packing and conventional surgical techniques when the patient is in extremis or when proximal vascular control is deemed necessary before opening the abdomen, aortic cross clamping may be employed as an adjunct to reduce bleeding and redistribute blood flow to the heart and brain [241-243]. When blood loss is significant, surgical measures are unsuccessful and/or when the patient is cold, acidotic and coagulopathic, definitive packing may also be the first surgical step within the concept of damage control [244-253].

Packing aims to compress liver ruptures or exert direct pressure on the sources of bleeding [239,240,244-248,250-252]. The definitive packing of the abdomen may allow further attempts to achieve total haemostasis through angiography and/or correction of coagulopathy [253]. The removal of packs should preferably be performed only after 48 h to lower the risk of re-bleeding [250,251]. Although clinical experience with the concept of damage control is good, the scientific evidence is limited [254].

#### Pelvic ring closure and stabilisation

##### Recommendation 19

We recommend that patients with pelvic ring disruption in haemorrhagic shock undergo immediate pelvic ring closure and stabilisation. (Grade 1B)

#### Packing, embolisation and surgery

##### Recommendation 20

We recommend that patients with ongoing haemodynamic instability despite adequate pelvic ring stabilisation receive early preperitoneal packing, angiographic embolisation and/or surgical bleeding control. (Grade 1B)

###### Rationale

The mortality rate of patients with severe pelvic ring disruptions and haemodynamic instability remains unacceptably high [255]. The early detection of these injuries and initial efforts to reduce disruption and stabilise the pelvis as well as contain bleeding is therefore crucial. Markers of pelvic hemorrhage include anterior-posterior and vertical shear deformations on standard roentgenograms, CT 'blush' (active arterial extravasation), bladder compression pressure, pelvic haematoma volumes >500 ml evident by CT and ongoing haemodynamic instability despite adequate fracture stabilisation [256,257].

The initial therapy of pelvic fractures includes control of venous and/or cancellous bone bleeding by pelvic closure. Some institutions use primarily external fixators to control hemorrhage from pelvic fractures [257], but pelvic closure may also be achieved using a bed sheet, pelvic binder or a pelvic C-clamp [258]. In addition to the pelvic closure, fracture stabilisation and the tamponade effect of the haematoma, pre-, extra- or retroperitoneal packing will reduce or stop the venous bleeding [259-262]. Pre-peritoneal packing decreases the need for pelvic embolisation and may be performed simultaneously or soon after initial pelvic fracture stabilisation. Pelvic packing could potentially aid in early intrapelvic bleeding control and provide crucial time for more selective management of hemorrhage [260,262]. The technique can be combined with a consecutive laparotomy if deemed necessary [259,260]. This may decrease the high mortality rate observed in patients with major pelvic injuries who underwent laparotomy as the primary intervention. As a consequence, it was recommended that non-therapeutic laparotomy be avoided [263].

Angiography and embolisation are currently accepted as a highly effective means with which to control arterial bleeding that cannot be controlled by fracture stabilisation [256-259,262-268]. Martinelli *et al*. [269] report on the use of intra-aortic balloon occlusion to reduce bleeding and permit transport to angiography. In contrast, Morozumi *et al*. [270] suggest the use of mobile digital subtraction angiography in the emergency department for arterial embolisation performed by trauma surgeons themselves. A number of authors stress that permissive hypotension while obtaining pelvic stabilisation and/or angiography (damage control resuscitation, hypertonic solutions, controlled hypothermia) could achieve better survival [170,271,272]. Controversy exists about the indications and optimal timing of angiography in haemodynamically unstable patients [262]. Institutional differences in the capacity to perform timely angiography and embolisation may explain the different treatment algorithms suggested by many authors [255,260,262,263,268,273,274]. Nevertheless, the general consensus is that a multidisciplinary approach to these severe injuries is required.

#### Damage control surgery

##### Recommendation 21

We recommend that damage control surgery be employed in the severely injured patient presenting with deep haemorrhagic shock, signs of ongoing bleeding and coagulopathy. (Grade 1B)

Other factors that should trigger a damage control approach are severe coagulopathy, hypothermia, acidosis, an inaccessible major anatomic injury, a need for time-consuming procedures or concomitant major injury outside the abdomen. (Grade 1C)

We recommend primary definitive surgical management in the haemodynamically stable patient and in the absence of any of the factors above. (Grade 1C)

###### Rationale

The severely injured patient arriving to the hospital with continuous bleeding or deep haemorrhagic shock generally has a poor chance of survival unless early control of bleeding, proper resuscitation and blood transfusion are achieved. This is particularly true for patients who present with uncontrolled bleeding due to multiple penetrating injuries or patients with major abdominal injury and unstable pelvic fractures with bleeding from fracture sites and retroperitoneal vessels. The common denominator in these patients is the exhaustion of physiologic reserves with resulting profound acidosis, hypothermia and coagulopathy, also known as the "bloody vicious cycle" or "lethal triad". In 1983, Stone described the techniques of abbreviated laparotomy, packing to control hemorrhage and of deferred definitive surgical repair until coagulation has been established [275]. Since then, a number of authors have described the beneficial results of this concept, now called "damage control" [249,276-278]. The type of multiply-injured patient who should be subjected to a damage control strategy is better defined today [279,280]. It should be considered in patients with major abdominal injury and a need for adjunctive use of angioembolisation, major abdominal injury and a need to evaluate early on other possible injuries, major abdominal injury and traumatic amputation of a limb. Factors that should trigger a damage control approach in the operating theatre are temperature ≤34°C, pH ≤7.2, an inaccessible major venous injury, a need for time-consuming procedures in a patient with suboptimal response to resuscitation or inability to achieve haemostasis due to recalcitrant coagulopathy.

Damage control surgery of the abdomen consists of three components. The first component is an abbreviated resuscitative laparotomy for control of bleeding, the restitution of blood flow where necessary and the control of contamination. This should be achieved as rapidly as possible without spending unnecessary time on traditional organ repairs that can be deferred to a later phase. The abdomen is packed and temporary abdominal closure is performed. The second component is intensive care treatment, focused on core re-warming, correction of the acid-base imbalance and coagulopathy as well as optimising the ventilation and the haemodynamic status. If complementary angiography and/or further injury investigation is needed, it should be performed. The third component is the definitive surgical repair that is performed only when target parameters have been achieved [63,249,276-278,281,282]. Although the concept of "damage control" intuitively makes sense, no RCTs exist to support it. Retrospective studies support the concept showing reduced morbidity and mortality rates in selective populations [278].

The same "damage control" principles have been applied to orthopaedic injuries in severely injured patients. Scalea *et al*. were the first to coin the term "damage control orthopaedics" [283]. Relevant fractures are primarily stabilised with external fixators rather than primary definitive osteosynthesis [265,283-285]. The less traumatic and shorter duration of the surgical procedure aims to reduce the secondary trauma load. Definitive osteosynthesis surgery can be performed after 4 to 14 days when the patient has recovered sufficiently. Retrospective clinical studies and prospective cohort studies seem to support the concept of damage control. The only available randomised study shows an advantage for this strategy in "borderline" patients [285]. The damage control concept has also been described for thoracic and neurosurgery as well as for post-traumatic anaesthesia [286-288].

#### Local haemostatic measures

##### Recommendation 22

We recommend the use of topical haemostatic agents in combination with other surgical measures or with packing for venous or moderate arterial bleeding associated with parenchymal injuries. (Grade 1B)

###### Rationale

A wide range of local haemostatic agents are currently available for use as adjuncts to traditional surgical techniques to obtain haemorrhagic control. These topical agents can be particularly useful when access to the site of bleeding is difficult. Local haemostatic agents include collagen, gelatine or cellulose-based products, fibrin and synthetic glues or adhesives that can be used for both external and internal bleeding while polysaccharide-based and inorganic haemostatics are still mainly used and approved for external bleeding.

The use of topical haemostatic agents should consider several factors, such as the type of surgical procedure, cost, severity of bleeding, coagulation status and each agent's specific characteristics. Some of these agents should be avoided when autotransfusion is used, and several other contraindications need to be considered [289,290]. The capacity of each agent to control bleeding was initially studied in animals but increasing experience in humans is now available [289-308].

The different types of local haemostatic agents are briefly presented below according to their basis and haemostatic capacity:

Collagen-based agents trigger platelet aggregation, resulting in clot formation when in contact with a bleeding surface. They are often combined with a pro-coagulant substance such as thrombin to enhance the haemostatic effect. A positive haemostatic effect has been shown in several human studies [291-294].

â€¢ Gelatine-based products can be used alone or in combination with a pro-coagulant substance [289]. Swelling of the gelatine in contact with blood reduces the blood flow and, in combination with a thrombin-based component, enhances haemostasis [295-297]. The products have been successfully used for local bleeding control in brain or thyroid surgery when electrocautery may cause damage to nerves [298] or to control bleeding from irregular surfaces, such as post-sinus surgery [299].

â€¢ The effect of cellulose-based haemostatic agents on bleeding has been less studied and only case reports that support their use are available.

â€¢ Fibrin and synthetic glues or adhesives have both haemostatic and sealant properties, and their significant effect on haemostasis has been shown in several human randomised controlled studies involving vascular, bone, skin and visceral surgery [300-302]

â€¢ Polysaccharide-based haemostatics can be divided into two broad categories [289]: *N*-acetyl-glucosamine-containing glycosaminoglycans purified from microalgae and diatoms and microporous polysaccharide haemospheres produced from potato starch. The mechanism of action is complex and depends on the purity or combination with other substances, such as cellulose or fibrin. A number of different products in the form of pads, patches or bandages are currently available and have been shown to be efficient for external use and for splanchnic bleeding in animals. An observational study showed that hemorrhage control was achieved using a poly-*N*-acetylglucosamine-based bandage applied to 10 patients with severe hepatic and abdominal injuries, acidosis and clinical coagulopathy [304].

â€¢ Inorganic haemostatics based on minerals, such as zeolite or smectite, have been used and studied mainly in the pre-hospital setting and on external bleeding sources [289,290].

### V. Management of bleeding and coagulation

#### Coagulation support

##### Recommendation 23

We recommend that monitoring and measures to support coagulation be initiated as early as possible. (Grade 1C)

###### Rationale

Major trauma results not only in bleeding from anatomical sites but frequently also in coagulopathy, which is associated with a several-fold increase in mortality [4,5,7,9,13,309]. This early coagulopathy of trauma is found mainly in patients with hypoperfusion (base deficit >6 mE/l) [9,309] and is characterised by an up-regulation of endothelial thrombomodulin, which forms complexes with thrombin [310].

Early monitoring of coagulation is essential to detect trauma-induced coagulopathy and to define the main causes, including hyperfibrinolysis [14,134,137,139,311,312]. Early therapeutic intervention does improve coagulation tests [313], reduce the need for transfusion of RBC, FFP and platelets [314,315], reduce the incidence of post-traumatic multi-organ failure [315], shorten length of hospital stay [314] and may improve survival [316,317]. Therefore, early aggressive treatment is likely to improve the outcome of severely injured patients [318]. However, there are also studies in which no survival benefit could be shown [313,319]; interestingly, in these studies only traditional lab values, such as PT, aPTT and platelet count, were used for coagulation monitoring and only FFP and platelets were used to treat coagulopathy.

#### Antifibrinolytic agents

##### Recommendation 24

We recommend that tranexamic acid be administered as early as possible to the trauma patient who is bleeding or at risk of significant hemorrhage at a loading dose of 1 g infused over 10 minutes, followed by an intravenous infusion of 1 g over 8 h. (Grade 1A)

We recommend that tranexamic acid be administered to the bleeding trauma patient within 3 h after injury. (Grade 1B)

We suggest that protocols for the management of bleeding patients consider administration of the first dose of tranexamic acid en route to the hospital. (Grade 2C)

###### Rationale

Tranexamic acid (trans-4-aminomethylcyclohexane-1-carboxylic acid; TXA) is a synthetic lysine analogue that is a competitive inhibitor of plasminogen. TXA is distributed throughout all tissues, and the plasma half-life is 120 minutes [320]. The CRASH-2 trial (Clinical Randomisation of Antifibrinolytic therapy in Significant Hemorrhage) [321] assessed the effects of early administration of a short course of TXA on the risk of death, vascular occlusive events and the receipt of blood product transfusion in trauma patients who were bleeding or at risk of significant bleeding. The trial randomised 20,211 adult trauma patients with or at risk of significant bleeding to either TXA (loading dose 1 g over 10 minutes followed by infusion of 1 g over 8 h) or matching placebo within 8 h of injury. The primary outcome was death in hospital within four weeks of injury. All analyses assessed the intention-to-treat population. All-cause mortality was significantly reduced with TXA (1,463 (14.5%) TXA vs. 1,613 (16.0%) placebo; relative risk 0.91, 95% CI 0.85 to 0.97; *P *= 0.0035), and the risk of death due to bleeding was significantly reduced (489 (4.9%) vs. 574 (5.7%); relative risk 0.85, 95% CI 0.76 to 0.96; *P *= 0.0077). There was no evidence that the effect of TXA on death due to bleeding varied by systolic blood pressure, Glasgow coma score or type of injury. The risk of precipitated thrombosis with the use of the lysine analogues TXA and ε-aminocaproic acid has been of major theoretical concern; however, CRASH-2 showed that the rate of thrombosis, especially myocardial infarction, was lower with the use of TXA. No adverse events were described with the use of TXA in CRASH-2, although an increased rate of seizures has been described in patients receiving a high dose of TXA when undergoing cardiac surgery [322].

A further analysis of the CRASH-2 data [323] showed that early treatment (≤1 h from injury) significantly reduced the risk of death due to bleeding (198/3,747 (5.3%) events TXA vs. 286/3,704 (7.7%) placebo; relative risk (RR) 0.68, 95% CI 0.57 to 0.82; *P *<0.0001). Treatment administered between 1 and 3 h also reduced the risk of death due to bleeding (147/3,037 (4.8%) vs. 184/2,996 (6.1%); RR 0.79, 0.64 to 0.97; *P *= 0.03). Treatment given after 3 h seemed to increase the risk of death due to bleeding (144/3,272 (4.4%) vs. 103/3,362 (3.1%); RR 1.44, 1.12 to 1.84; *P *= 0.004), therefore, we recommend that TXA not be given more than 3 h following injury. In order to ensure that TXA is given early, the administration of TXA at the pre-hospital site of injury needs to be planned, and we suggest that protocols for the management of bleeding patients consider administration of the first dose of TXA at the site of injury. Left to clinical judgment for those at "high risk" or use only in massive blood loss protocols receiving TXA, it is estimated that only 40% of these deaths arise from the high risk patient group [324]. For a larger impact, TXA should be administered to all patients with trauma and significant bleeding. Thus guidelines for managing "massive blood loss" may need to be revised to include all patients who are bleeding, not just those with major hemorrhage.

The cost-effectiveness of TXA in trauma has been calculated in three countries [325]: Tanzania as an example of a low-income country, India as a middle-income country and the UK as a high-income country. The cost of TXA administration to 1,000 patients was US$17,483 in Tanzania, US$19,550 in India and US$30,830 in the UK. The estimated incremental cost per life year gained of administering TXA is $48, $66 and $64 in Tanzania, India and the UK, respectively.

ε-aminocaproic acid is also a synthetic lysine analogue that has a potency 10-fold weaker than that of TXA. It is administered at a loading dose of 150 mg/kg followed by a continuous infusion of 15 mg/kg/h. The initial elimination half-life is 60 to 75 minutes and must, therefore, be administered by continuous infusion in order to maintain therapeutic drug levels until the bleeding risk has diminished. This agent is a potential alternative to TXA if TXA is not available.

The use of aprotinin is contraindicated in bleeding trauma patients, now that TXA has been shown to be efficacious and safe in trauma, and there have been concerns about the safety of aprotinin in other settings [326].

#### Calcium

##### Recommendation 25

We recommend that ionised calcium levels be monitored and maintained within the normal range during massive transfusion. (Grade 1C)

###### Rationale

Two recent observational cohort studies have shown that low ionised calcium levels at admission are associated with an increased mortality as well as an increased need for massive transfusion [327,328]. Moreover, hypocalcaemia during the first 24 h can predict mortality and the need for multiple transfusion better than the lowest fibrinogen concentrations, acidosis and the lowest platelet counts [327]. Measurement of ionised calcium levels at admission may facilitate the rapid identification of patients requiring massive transfusion, allowing for earlier preparation and administration of appropriate blood products. However, no data are available to demonstrate that the prevention of ionised hypocalcaemia can reduce mortality among patients with critical bleeding requiring massive transfusion.

Calcium in the extracellular plasma exists either in a free ionised state (45%) or bound to proteins and other molecules in a biologically inactive state (55%). The normal concentration of the ionised form ranges from 1.1 to 1.3 mmol/l and is influenced by the pH. A 0.1 unit increase in pH decreases the ionised calcium concentration by approximately 0.05 mmol/l [329]. The availability of ionised calcium is essential for the timely formation and stabilisation of fibrin polymerisation sites, and a decrease in cytosolic calcium concentration precipitates a decrease in all platelet-related activities [330]. In addition, contractility of the heart and systemic vascular resistance are low at reduced ionised calcium levels. Combining beneficial cardiovascular and coagulation effects, the level for ionised calcium concentration should, therefore, be maintained >0.9 mmol/l [330].

Early hypocalcaemia following traumatic injury shows a significant correlation with the amount of fresh frozen plasma transfused and also with the amount of infused colloids, but not with crystalloids. Hypocalcaemia develops during massive transfusion as a result of the citrate employed as an anticoagulant in blood products. Citrate exerts its anticoagulant activity by binding ionised calcium, and hypocalcaemia is most common in association with FFP and platelet transfusion because these products contain high citrate concentrations. Citrate undergoes rapid hepatic metabolism, and hypocalcaemia is generally transient during standard transfusion procedures. Citrate metabolism may be dramatically impaired by hypoperfusion states, hypothermia and in patients with hepatic insufficiency [330].

#### Plasma

##### Recommendation 26

**We recommend the initial administration of plasma (fresh frozen plasma (FFP) or pathogen-inactivated plasma) (Grade 1B) or fibrinogen (Grade 1C) in patients with massive bleeding**.

If further plasma is administered, we suggest an optimal plasma:red blood cell ratio of at least 1:2. (Grade 2C)

We recommend that plasma transfusion be avoided in patients without substantial bleeding. (Grade 1B)

###### Rationale

Damage control resuscitation aims to rapidly address acute traumatic coagulopathy through the early replacement of clotting factors. Plasma (thawed FFP or pathogen-inactivated plasma/industrial purified plasma) is used throughout the world as a source of fibrinogen and clotting factors. FFP has about 70% of the normal level of all clotting factors; therefore, it seems to be an adequate source for replacement; however, different preparations show great variability [331]. Acidosis as a consequence of massive hemorrhage has a detrimental effect on the coagulation cascade; a low pH strongly affects the activity of factor VII and to a lesser extent factor × and factor V [272]. Moreover, recent studies demonstrated that hypoperfusion in trauma patients is associated with an early and marked reduction in factor V activity and with a less important decrease in the activity of factors II, VII, IX, × and XI [332]. The marked fall in factor V probably represents fibrinolytic activation because factor V is very susceptible to breakdown by fibrinolysis [333]. Trauma-associated coagulopathy is present in as many as 25% to 30% of patients with major trauma [6,7] on arrival in the emergency department.

The use of plasma is not hazard-free and is associated with an increased incidence of post-injury multiple organ failure [334-336], acute respiratory distress syndrome (ARDS) [334,337], infections [334,338] and with an increasing complication rate as the volume of plasma increases [335,336]. As with all products derived from human blood, the risks associated with FFP treatment also include circulatory overload, ABO incompatibility, transmission of infectious diseases (including prion diseases) and mild allergic reactions. Transfusion-related acute lung injury (TRALI) [339,340] is a severe adverse effect associated with the presence of leucocyte antibodies in transfused plasma. Transmission of infectious diseases can be minimised by the use of pathogen-inactivated plasma (industrial purified plasma).

Although the formal link between the administration of FFP, control of bleeding and an improvement in the outcome of bleeding patients is lacking, some experts would agree that FFP treatment is beneficial in patients with massive bleeding or significant bleeding complicated by coagulopathy. Based on reports from the Iraq War, in May 2005 an international expert conference on massive transfusion at the US Army's Institute of Surgical Research introduced a new concept for resuscitation of patients with massive bleeding and recommended the immediate administration of coagulation components with a 1:1:1 ratio for RBC, plasma and platelets [341-343]. In the following few years retrospective evidence from both military and civilian practice suggested improved outcomes in patients with massive bleeding after the adoption of a massive transfusion protocol, including the early administration of high-dose plasma therapy [344]. In the meantime, 19 studies [135,313,316,319,345-359], 6 systematic reviews [360-365] and 1 meta-analysis [366] have addressed the impact of FFP:RBC ratio. However, these studies have severe limitations: none are RCTs, all but three [319,348,359] are retrospective and the majority have a number of potential confounders that might introduce relevant bias. The majority of the authors used massive transfusion (10 RBC units within 24 h) as the entry criterion, but Davenport *et al*. [359] focused on significant bleeding (>4 units RBC), Borgman *et al*. [358] used the TASH score to identify patients who would need a high FPP:RBC ratio, while other authors used a different time span than 24 h. A significant heterogeneity among the different studies is, therefore, present. Moreover, FFP needs to be thawed before administration; therefore it is often not immediately available. As 50% of patients who die because of hemorrhage die within the first 6 h, many of them might not live long enough to receive blood products at the intended ratio, introducing potential time and survival biases that may contribute to confounding results [277,352,356,357]. To avoid this bias some investigators have excluded those patients who died within a few hours of hospital admission, but this may introduce a different but relevant bias because patients who died from exanguination, but could have benefited from a higher plasma:RBC ratio, are not included in these analyses [367,368]. For all of these reasons, the quality of evidence is very low. In general, outcomes were favourable for patients who received a higher plasma:RBC ratio; however, the optimal ratio required to achieve an improvement in the survival rate was not consistent. The single meta-analysis [366] showed a significant reduction in the risk of death (OR 0.38, CI 0.24 to 0.60) for trauma patients undergoing massive transfusion at a plasma:RBC ratio in the range of 1:2.5 to 1:1, but the authors caution against the very low level of supporting evidence. The majority of the systematic reviews reached the same conclusions, suggesting an improved mortality with a higher level of plasma [360-363], though emphasising that an optimal and consistent FFP:RBC ratio has not yet been identified [365], and there is insufficient evidence to support the use of a fixed 1:1 ratio [362]. Lier *et al*. [363] were the only author group who felt that the evidence was strong enough to suggest that a ratio of 1:2 to 1:1 FFP:RBC should be targeted. In contrast, a review by Kozek *et al*. [364] reached the conclusion that there is inconsistent and contradictory evidence concerning the efficacy of FFP, and suggested that fibrinogen might offer some alternative advantage, although high-quality prospective studies are required before any conclusion can be drawn.

Interestingly, a recent prospective cohort study by Davenport *et al*. [359] analysed coagulation parameters before and after transfusion of every four units of RBC with variable rates of plasma by rotational thromboelastometry. These authors observed a maximal haemostatic effect with a plasma:RBC ratio ranging between 1:2 and 3:4. A higher rate did not bring any additional improvement, and in some patients the haemostatic function deteriorated. These data are consistent with the results of computer-generated models of massive transfusion [277].

#### Fibrinogen and cryoprecipitate

##### Recommendation 27

We recommend treatment with fibrinogen concentrate or cryoprecipitate in the continuing management of the patient if significant bleeding is accompanied by thromboelastometric signs of a functional fibrinogen deficit or a plasma fibrinogen level of less than 1.5 to 2.0 g/l. (Grade 1C)

We suggest an initial fibrinogen concentrate dose of 3 to 4 g or 50 mg/kg of cryoprecipitate, which is approximately equivalent to 15 to 20 single donor units in a 70 kg adult. Repeat doses may be guided by viscoelastic monitoring and laboratory assessment of fibrinogen levels. (Grade 2C)

###### Rationale

Fibrinogen is the final component in the coagulation cascade, the ligand for platelet aggregation and, therefore, key to effective coagulation and platelet function [233,369]. Hypofibrinogenemia is a usual component of complex coagulopathies associated with massive bleeding. Coagulopathic civilian trauma patients had a fibrinogen concentration of 0.9 g/l (interquartile ratio (IQR) 0.5 to 1.5 g/l) in conjunction with a maximum clot firmness (MCF) of 6 mm (IQR 0 to 9 mm) using thromboelastometry, whereas only 2.5% of healthy volunteers had a MCF of <7 mm [14]. In trauma patients, a MCF of 7 mm was associated with a fibrinogen level of approximately 2 g/l [14]. During massive blood loss replacement, fibrinogen is the first coagulation factor to critically decrease [370]. During postpartum hemorrhage, fibrinogen plasma concentration is the only coagulation parameter independently associated with progress toward severe bleeding, with a level <2 g/l having a positive predictive value of 100% [371].

An early observational study suggested that fibrinogen substitution can improve survival in combat-related trauma [372]. Subsequent retrospective reviews of single centre experiences managing massive blood loss in trauma patients have suggested that the use of thromboelastometry-guided fibrinogen with other blood products reduced mortality when compared to expected mortality [317], reduced the exposure to allogeneic blood products [314] and increased 30-day survival [355]. However, as recent systematic reviews have shown [364,373], there are no adequately powered prospective clinical trials to demonstrate the risk:benefit analysis of using a source of additional fibrinogen to manage bleeding trauma patients.

Fibrinogen administration using viscoelastic methods as guidance may be preferable to measuring fibrinogen levels in the laboratory. Some methodological issues in the various laboratory methods to measure fibrinogen concentration remain [374,375], and in the presence of artificial colloids, such as HES, even the most frequently recommended method [374], the Clauss method, significantly overestimates the actual fibrinogen concentration [375].

The issue of whether the administration of fibrinogen via factor concentrate, cryoprecipitate or FFP is associated with an increased risk of hospital-acquired venous thromboembolism has never been addressed. However, fibrinogen levels are expected to rise to a level of approximately 7 g/l after major surgery and trauma [376,377] even without intra-operative fibrinogen administration, and the effect of intra-operative fibrinogen administration on post-traumatic fibrinogen levels are unknown. Interestingly, intra-operative administration of fibrinogen concentrate in patients undergoing cystectomy and cardiac surgery resulted in higher early postoperative fibrinogen levels but already at 24 h post-operation fibrinogen levels were identical in patients with and without intra-operative fibrinogen administration [378,379]. Well-designed prospective, randomised double-blinded studies evaluating the effect of fibrinogen supplementation are urgently needed.

The rationale for fibrinogen administration should be read in conjunction with that for plasma (R26).

#### Platelets

##### Recommendation 28

We recommend that platelets be administered to maintain a platelet count above 50 × 10^9^/l. (Grade 1C)

We suggest maintenance of a platelet count above 100 × 10^9^/l in patients with ongoing bleeding and/or TBI. (Grade 2C)

We suggest an initial dose of four to eight single platelet units or one aphaeresis pack. (Grade 2C)

###### Rationale

The role of platelets in the development of traumatic coagulopathy is not fully understood; however, there is weak scientific evidence to support a particular platelet transfusion threshold in the trauma patient. One small prospective study performed in massively transfused patients found a platelet count of <100 × 10^9^/l as the threshold for diffuse bleeding [380], and another study indicated a platelet count <50 × 10^9^/l or fibrinogen <0.5 g/l as the most sensitive laboratory predictors of microvascular bleeding [381]. Patients with both platelet and fibrinogen values above these levels had only a 4% chance of developing microvascular bleeding. A platelet count >100 × 10^9^/l further improved survival in patients with massive bleeding due to ruptured aortic abdominal aneurysms treated proactively with platelet transfusion compared to lower levels [382].

As a result, expert consensus is that the platelet count should not be less than the critical level of 50 × 10^9^/l in the acutely bleeding patient [383], with some experts claiming that a higher threshold of 75 × 10^9^/l provides a margin of safety [384,385]. A higher target level of 100 × 10^9^/l has been suggested for those with multiple trauma, brain injury and massive bleeding [383,384]. Recently, it was found that a platelet count of <100 × 10^9^/l was an independent predictor of mortality in patients with TBI [386].

Furthermore, in most trauma patients, the admission platelet count is within the normal range [387-389], with less than 5% of patients arriving in the emergency room with a platelet count of <100 × 10^9^/l [7]. In initial acute loss, the bone marrow and spleen variably release platelets, and a platelet count of 50 × 10^9^/l may be anticipated when approximately two blood volumes have been replaced by fluid or red cell components [370]. In addition, in patients exhibiting traumatic coagulopathy, the platelet count does not decline to levels that might be expected to contribute significantly to coagulopathy [389]. However, the platelet count on admission, may be predictive of outcome as documented in some cohorts of massively transfused trauma patients, where platelet count was inversely correlated with injury severity [387], morbidity [386] and mortality [387,388,390].

Thus, a normal platelet count may be insufficient after severe trauma, and platelet count alone is a weak indicator of platelet transfusion needs because it ignores platelet dysfunction. Additionally, platelet function in trauma patients has been poorly investigated. Severe injury can result in increased platelet activation, which, along with decreased function as observed in TBI, was associated with increased mortality [391]. Similarly, non-survivors in a small study had minor but significantly more platelet defects as assessed by multiplate electrode aggregometry compared to survivors [160]. Recently it was found that after an injury the platelet dysfunction is present even before substantial fluid or blood transfusion takes place and continues during the resuscitation period, this suggesting a potential role for early platelet transfusion [392].

The normal therapeutic dose of platelets is one concentrate (60 to 80 × 10^9 ^platelets) per 10 kg body weight. One aphaeresis platelet product, which is approximately equivalent to six whole blood-derived units, generally contains approximately 3 to 4 × 10^11 ^platelets in 150 to 450 ml donor plasma [383,385], depending on local collection practice. A dose of four to eight platelet units or a single-donor aphaeresis unit is usually sufficient to provide haemostasis in a thrombocytopenic, bleeding patient and should increase the platelet count by 30 to 50 × 10^9^/l [393]. The platelet concentrate transfused must be ABO-identical, or at least ABO-compatible, in order to provide a good yield [385].

For the management of traumatic coagulopathy, there is still no high-quality evidence supporting up-front platelet transfusion or higher doses given in pre-defined ratios with other blood products. The only prospective randomised trial evaluating prophylactic platelet transfusion at a ratio to whole blood of 1:2 versus the same amount of plasma in patients receiving ≥12 units of whole blood in 12 h concluded that platelet administration did not affect microvascular non-surgical bleeding [394]. Although most of the further studies [348,349,354,395-397] and a meta-analysis including studies published between 2005 and 2010 [398] that investigated the impact of platelet transfusion in severe trauma and massive transfusion showed an improved survival rate among patients receiving high platelet:RBC ratios, such evidence provided by retrospective and observational studies may be subject to serious confounding factors, such as survivorship bias. The timing of platelet transfusion in relation to the initiation of RBC/FFP transfusion was not reported in most of the studies, and there might be more than one optimal ratio according to trauma severity, degree and dynamics of blood loss and previous fluid administration [398]. A recent analysis of a large prospective cohort showed that high platelet:RBC ratio was associated with survival benefit as early as 6 h and throughout the first 24 h, even when time-dependent fluctuations in component transfusion are accounted for, suggesting that survivor bias is unlikely [399]. Negative [400,401] and partially positive results [402] were also reported in patients with massive transfusion. Interestingly, patients with penetrating injuries [400] and females [402] do not benefit from high platelet:RBC ratios, and no difference in mortality was observed in patients with non-massive transfusion receiving higher platelet:RBC ratios [403]. When a research intervention (before-and-after introduction of a massive hemorrhage protocol performed with high plasma and platelet:RBC ratios) was reported, improved survival was shown in three studies [135,344,355], but not in a further study [404]. Therefore, the administration of high platelet:RBC ratios along with high plasma:RBC ratios remains controversial.

One additional reason for the lack of clarity is the difficulty in separating the effect of a high platelet:RBC ratio from the effect of a high plasma:RBC ratio. Patients receiving a combination of high plasma and high platelet ratios had an improved 6-h [349,354,399], 24-h [344,349,395-397,399], 30-day [135,344,348,349,355,395,397], in hospital [354] and discharge survival [396]. However, the impact exerted by platelets on survival was not as strong as that of plasma transfusion [348,396], higher than the impact of plasma [355] or even absent, in contrast to the benefit of increased plasma:RBC ratios [401]. On the contrary, transfusion of a high platelet:RBC ratio and not a high plasma:RBC ratio was found to be associated with improved survival in patients with TBI [405].

One major drawback to these observational studies is the wide range of platelet:RBC ratios, along with reported poor compliance with specified platelet ratios during active resuscitation [406]. As a result, the definition of the optimal ratio of platelet:RBC transfusion remains elusive. A potential shortcoming of ratio-driven blood support is over-transfusion with plasma and platelets, resulting in no benefit or in added morbidity, such as multiple organ failure [334]. The age of transfused platelets may also play a role [407]. Although decreased morbidity due to aggressive use of plasma and platelets has been reported [318,349,354], routine early prophylactic platelet transfusion as part of a massive transfusion protocol appears unjustified at this time

#### Antiplatelet agents

##### Recommendation 29

We suggest administration of platelets in patients with substantial bleeding or intracranial hemorrhage who have been treated with antiplatelet agents. (Grade 2C)

If the patient has been treated with acetylsalicylic acid alone, we suggest administration of desmopressin (0.3 µg/kg). (Grade 2C)

We suggest the measurement of platelet function in patients treated or suspected of being treated with antiplatelet agents. (Grade 2C)

If platelet dysfunction is documented in a patient with continued microvascular bleeding, we suggest treatment with platelet concentrates. (Grade 2C)

###### Rationale

Little is known about the effects of antiplatelet agents (APAs), mainly aspirin and clopidogrel, on traumatic bleeding. Data from non-elective orthopaedic procedures show both increased peri-operative blood loss in patients taking APAs before surgery [408,409] or no effect [410]. The increase in blood transfusion in orthopaedic patients on APAs is also controversial [410,411]. However, the pre-injury use of APAs did not affect morbidity and mortality in retrospective studies in patients with pelvic fractures [412] or general trauma without brain injury [413], but did have an effect in patients with hip fractures [409]. On the contrary, even mild head trauma (GCS 14 to 15) while on APAs is associated with a high incidence of intracranial hemorrhage (ICH) [414-416], and a risk of delayed ICH in this group of patients mandates a longer period of observation [417,418]. Moreover, observational studies found a five-fold increase in traumatic ICH in patients on APAs [419]. The relationship between outcome and pre-injury APAs in the setting of ICH is conflicting in both the trauma [420-424] and stroke literature [425-427], and a systematic review of the latter has shown that pre-ICH APA users experienced only modestly increased mortality (OR 1.27; 95% CI 1.10 to 1.47) and little or no increase in poor clinical functional outcome (OR 1.10; 95% CI 0.93 to 1.29) [428].

Few studies have directly focused on outcome associated with a specific APA. Those that have analysed the use of clopidogrel prior to both spontaneous and traumatic ICH reported worsened outcome [426,429,430]. Compared to controls, patients on clopidogrel demonstrated a 14.7-fold increase in mortality [430], increased morbidity [429] and a 3-fold increase in disposition to a long-term facility [430]. On the contrary, pre-injury aspirin did not affect outcomes in mild to moderate head injury [431] or mortality [432]. Surprisingly, reduced platelet activity has been shown in patients with ICH in the absence of known aspirin use [433], and this was associated with more ICH volume growth and worse three-month outcome [434]. Early platelet dysfunction was also prevalent after severe TBI in the absence of APAs [435]. However, greater platelet inhibition was identified among patients taking a combination of APAs compared to those on single agents [433]. These findings coupled with the fact that 20 to 30% of patients are non-responders to aspirin, clopidogrel or both agents [436] suggest that reliable measures of platelet function would be useful in the setting of the bleeding trauma patient to guide clinicians on the use of platelet transfusion or other reversal agents. Patients with occult platelet dysfunction could be identified and unnecessary platelet transfusion could be avoided [432].

Currently, there is no agreement on the optimal assay for platelet function, and controversy exists as to whether ICH in the setting of APAs use warrants platelet transfusion. Transfusion of platelets has a low grade recommendation in the guidelines on ICH management in patients on APAs [437] and is currently indicated for patients on clopidogrel and traumatic hemorrhage, although its clinical utility remains to be established [438]. Retrospective studies have failed to show an outcome benefit from platelet transfusion in patients on APAs with spontaneous [427,439] or traumatic ICH [421,440,441]. A meta-analysis of six small studies of the impact of platelet transfusion on survival in patients with pre-injury APAs who experienced ICH, either spontaneous or traumatic, found no clear benefit [442]. Similarly, a systematic review of five retrospective registry studies on traumatic ICH provides inadequate evidence to support the routine use of platelet transfusion in patients with pre-injury antiplatelet use [443]. However, the timing of platelet administration was not optimal in some studies [434,439], and a small prospective study showed that early platelet transfusion, within 12 h of symptom onset, improved platelet activity and was associated with smaller final hemorrhage size and more independence at three months [444]. Another explanation for the observation that platelet transfusion shows no obvious benefit is that the inhibitory effect of the APAs is not being normalised due to insufficient dose or recent ingestion of APAs, which may inactivate transfused platelets [444]. The results of a multi-centre RCT on platelet transfusion in patients with APA-associated ICH are awaited [445].

The suggested dose for normalising platelet activity in healthy volunteers given aspirin alone or a combination of aspirin and clopidogrel was 5 and 10 to 15 platelet units, respectively [446]. Successful peri-operative management of patients on aspirin and clopidogrel requiring urgent surgery using two apheresis platelet units was recently reported [447]. Given that an active metabolite of clopidogrel persists after cessation of the medication, and that the half-life of transfused platelets is short, recurring platelet transfusion may be justified [448].

Besides platelet transfusion, current potential antiplatelet reversal therapies include desmopressin and recombinant activated coagulation factor VII (rFVIIa) [438]. The clinical utility of desmopressin and rFVIIa has not been assessed for reversal of the effects of pre-injury APAs in patients with traumatic ICH. Although desmopressin has been shown to improve platelet function in volunteers on aspirin [449] and clopidogrel [450], and peri-operatively in patients with mild inherited platelet defects [451], the use of desmopressin for acquired bleeding disorders is not supported by sound clinical evidence. One older meta-analysis suggested a benefit of desmopressin in patients taking aspirin [452], and desmopressin has been recommended in patients taking platelet inhibitors and suffering from ICH [438,453]. The standard dose is 0.3 µg/kg diluted in 50 ml saline and infused over 30 minutes [451]. Recently, it was shown that identification of impaired platelet function with a platelet function analyzer PFA-100 [454] or whole blood multiple electrode aggregometer [455] might be helpful in the identification of patients who may benefit from desmopressin therapy. The combined effect of platelet concentrates and subsequent administration of desmopressin has also been advocated to enhance the recovery of normal platelet function [456]. Furthermore, rFVIIa reversed the inhibitory effects of aspirin and clopidogrel in healthy volunteers [457]. Interestingly, the effective dose was lower than the dose used in haemophilia patients [458]. In addition, TXA was shown to partially improve platelet function in patients treated with dual antiplatelet therapy as measured by multiple electrode aggregometry [459]. Potential effectiveness in improving haemostasis in trauma patients receiving APAs was also shown for fibrinogen concentrate [460].

#### Desmopressin

##### Recommendation 30

We suggest that desmopressin (0.3 µg/kg) be administered in patients treated with platelet-inhibiting drugs or with von Willebrand disease. (Grade 2C)

We do not suggest that desmopressin be used routinely in the bleeding trauma patient. (Grade 2C)

###### Rationale

Desmopressin (DDAVP; 1-deamino-8-D-arginine vasopressin) enhances platelet adherence and platelet aggregate growth on human artery subendothelium and is the first choice in the treatment of bleeding in patients with von Willebrand disease, a disease which occurs roughly in 1 in 100 patients [461,462]. Two meta-analyses in patients not diagnosed with von Willebrand disease [463,464] were able to demonstrate either a trend towards a reduced peri-operative blood loss [463] or a small significant reduction in blood transfusion requirements (-0.29 (-0.52 to -0.06) units per patient) [464]. Patients with impaired platelet function as assessed by a platelet function analyser [454] or whole blood multiple electrode aggregometer [455] may benefit from desmopressin therapy. Concerns regarding possible thromboembolic complications [465] were not confirmed in the last meta-analysis from 2008 [464].

Desmopressin has never been formally investigated in general trauma or TBI [438]. Nevertheless, desmopressin has been recommended in patients treated with platelet inhibitors, suffering from intracerebral bleeding [438,453] and in trauma patients with von Willebrand disease [466]. Interestingly, desmopressin prevents the development of hypothermia-induced impairment of primary haemostasis [467] and significantly increases platelet aggregation during hypothermia and acidosis [468].

#### Prothrombin complex concentrate

##### Recommendation 31

We recommend the early use of prothrombin complex concentrate (PCC) for the emergency reversal of vitamin K-dependent oral anticoagulants. (Grade 1B)

If a concentrate-based goal-directed strategy is applied, we suggest that PCC be administered in the bleeding patient with thromboelastometric evidence of delayed coagulation initiation. (Grade 2C)

###### Rationale

Despite the increasing use of PCC, including activated PCC, there are no large RCTs to support its use other than in haemophilia [469-471] or for the rapid reversal of the effect of oral vitamin K antagonists [472-474]. In the setting of trauma patients treated with pre-injury warfarin, a retrospective analysis showed that the use of PCC resulted in a more rapid time to reversal of the INR [475-478]. Thromboelastometry appears to be a useful tool to guide PCC therapy in patients with traumatic coagulopathy [314,475,479-482]. With an ageing population, more trauma patients are likely to have been pre-treated with vitamin K antagonists; therefore, every trauma unit should have an established management policy for these patients [476]. Because there are variations in the production of PCC, the dosage should be determined according to the instructions of the individual manufacturer [483,484].

The use of PCC carries the increased risks of both venous and arterial thrombosis during the recovery period; therefore, the risk of a thrombotic complication due to treatment with PCCs should be weighed against the need for rapid and effective correction of coagulopathy [485-488]. Thromboprophylaxis as early as possible after control of bleeding has been achieved is recommended in patients who have received PCC.

#### Novel anticoagulants

##### Recommendation 32

We suggest the measurement of substrate-specific anti-factor Xa activity in patients treated or suspected of being treated with oral anti-factor Xa agents such as rivaroxaban, apixaban or endoxaban. (Grade 2C)

If bleeding is life-threatening, we suggest reversal of rivaroxaban, apixaban and endoxaban with high-dose (25 to 50 U/kg) PCC. (Grade 2C)

We do not suggest the administration of PCC in patients treated or suspected of being treated with oral direct thrombin inhibitors, such as dabigatran. (Grade 2B)

###### Rationale

In recent years, new oral anticoagulants for the prevention of venous thromboembolism, prevention of stroke in atrial fibrillation, reduction of cardiovascular events in patients with acute coronary syndrome and treatment of pulmonary embolism and deep venous thrombosis (DVT) have been developed. The primary modes of action by these novel drugs are direct factor Xa inhibition (rivaroxaban, apixaban and endoxaban) or thrombin inhibition (dabigatran) [489]. We are, therefore, increasingly likely to be confronted with trauma patients who have been treated with one of these drugs [490], which exert an effect on both coagulation tests [490,491] and haemostasis [492].

No published clinical studies and very little clinical experience in traumatically injured patients who have been treated with one of these drugs exist [491,493]. However, it was recently shown that the effect of these drugs on coagulation tests of factor Xa (rivaroxaban) but not of factor IIa (dabigatran) antagonists in human volunteers could be immediately and completely reversed with high-dose (50 U/kg) PCC [494].

Anti-factor Xa activity can be measured with a substrate-specific anti-factor Xa test in trauma patients known or suspected to have been treated with factor Xa antagonists. If anti-factor Xa activity is detected, high-dose (25 to 50 U/kg) PCC treatment may be initiated. We suggest an initial dose of 25 U/kg, repeated if necessary, as a cautious approach given the possible thrombotic potential of PCC products [486]. Factor IIa antagonist treatment does prolong aPTT and thrombin time but high-dose (50 U/kg) PCC treatment is inefficient [494]. Aside from a consideration of haemodialysis [495] or the administration of factor VIII inhibitor bypassing activity [496], no specific treatment for patients treated with a factor IIa antagonist can be recommended at the current time. The involvement of a haematologist with expertise in coagulation should be considered.

#### Recombinant activated coagulation factor VII

##### Recommendation 33

We suggest that the use of recombinant activated coagulation factor VII (rFVIIa) be considered if major bleeding and traumatic coagulopathy persist despite standard attempts to control bleeding and best-practice use of conventional haemostatic measures. (Grade 2C)

We do not suggest the use of rFVIIa in patients with intracerebral hemorrhage caused by isolated head trauma. (Grade 2C)

###### Rationale

rFVIIa is not a first-line treatment for bleeding and can be effective only once sources of major bleeding have been controlled. Once major bleeding from damaged vessels has been stopped, rFVIIa may be helpful to induce coagulation in areas of diffuse small vessel coagulopathic bleeding. rFVIIa should be considered only if first-line treatment with a combination of surgical approaches, best-practice use of blood products, (RBC, platelets, FFP and cryoprecipitate/fibrinogen resulting in Hct above 24%, platelets above 50 × 10^9^/l and fibrinogen above 1.5 to 2.0 g/l), the use of antifibrinolytics and correction of severe acidosis, severe hypothermia and hypocalcaemia fail to control bleeding.

Because rFVIIa acts on the patient's own coagulation system, adequate numbers of platelets and fibrinogen levels are needed to allow a thrombin burst to be induced by the pharmacological, supra-physiological doses of rFVIIa through direct binding to activated platelets [497,498]. pH and body temperature should be restored as near to physiological levels as possible, since even small reductions in pH and temperature result in slower coagulation enzyme kinetics [196,197,499]. Predictors of a poor response to rFVIIa were a pH <7.2 (*P *<0.0001), a platelet count <100 × 10^9^/l (*P *= 0.046), and blood pressure ≤90 mmHg (*P *<0.0001) at the time of administration of rFVIIa [500]. Moreover, hypocalcaemia is frequently present in severely injured patients [501]; therefore, monitoring of ionised calcium is necessary, and administration of intravenous calcium may be required [502].

Despite numerous case studies and series reporting that treatment with rFVIIa can be beneficial in the treatment of bleeding following trauma, there are few high quality studies [503-506]. A multi-centre, randomised, double-blind, placebo-controlled study examined the efficacy of rFVIIa in patients with blunt (n = 143) or penetrating (n = 134) trauma [507] and showed that patients with blunt trauma who survived for more than 48 h assigned to receive rFVIIa 200 µg/kg after they had received eight units of RBC and a second and third dose of 100 µg/mg 1 and 3 h later had a reduction in RBC transfusion requirements and the need for massive transfusions (>20 units of RBC) compared to placebo. They also had a significantly reduced incidence of ARDS. In contrast, there were no significant effects in the penetrating trauma patients in this study, although trends toward reduced RBC requirements and fewer massive transfusions were observed. Similar results and trends were observed in other retrospective studies and case reports [508-510]. A further randomised clinical trial [511] aimed to evaluate rFVIIa as an adjunct to direct haemostasis in major trauma patients who bled four to eight RBC units within 12 h of injury and were still bleeding despite strict damage control resuscitation and operative management. Patients were treated with rFVIIa (200 µg/kg initially; 100 µg/kg at 1 and 3 h) or placebo. The trial was terminated early (n = 573) due to difficulty in consenting and enrolling sicker patients and resulting low mortality rates that prompted a futility analysis. Thrombotic adverse events were similar across study cohorts.

In contrast, the use of rFVIIa in isolated head injury was found to be harmful in a case-controlled study of patients with traumatic intracranial hemorrhage, with the risk of death appearing to increase with administration regardless of the severity of injury [512]. No reliable evidence from RCTs exists to support the effectiveness of haemostatic drugs in reducing mortality or disability in patients with TBI [513].

The required dose(s) of rFVIIa is still under debate. Whereas the dosing used in the published RCTs in trauma patients is also recommended by a group of European experts [514], Israeli guidelines based on findings from a case series of 36 patients who received rFVIIa on a compassionate-use basis [504] propose an initial dose of 120 µg/kg (between 100 and 140 µg/kg) and (if required) a second and third dose. Pharmacokinetic modelling techniques have shown that the dose regimen for rFVIIa treatment used in the RCT described above is capable of providing adequate plasma levels of drug to support haemostasis [515].

If rFVIIa is administered, the patient's next of kin should be informed that rFVIIa is being used outside the currently approved indications (off-label use), especially since the use of rFVIIa may increase the risk of thromboembolic complications [516]. A meta-analysis performed by the manufacturer showed a higher risk of arterial thrombomebolic adverse events (5.6% in patients receiving rFVIIa versus 3.0% in placebo-treated patients) among over 2,000 patients enrolled in placebo-controlled trials outside currently approved indications in various clinical settings [517]. In trauma patients, however, rFVIIa use was not associated with an increased risk of thromboembolic complications [518].

#### Thromboprophylaxis

##### Recommendation 34

We suggest mechanical thromboprophylaxis with intermittent pneumatic compression (IPC) and/or anti-embolic stockings as soon as possible. (Grade 2C)

We recommend pharmacological thromboprophylaxis within 24 h after bleeding has been controlled. (Grade 1B)

We do not recommend the routine use of inferior vena cava filters as thromboprophylaxis. (Grade 1C)

###### Rationale

The risk of hospital-acquired venous thromboembolism is high after multiple trauma, exceeding 50%; pulmonary embolism is the third leading cause of death in those who survive beyond the third day [519]. There are few RCTs investigating thromboprophylaxis in trauma patients, and the use of anti-embolic stockings has never been evaluated in trauma patients. A meta-analysis was unable to show any reduction in the rate of DVT with intermittent pneumatic compression (IPC) [520]; however, mechanical methods are widely used because of the low bleeding risk.

The same meta-analysis showed that low-dose unfractionated heparin (LDUH) was no more effective than no thromboprophylaxis [520]. A large RCT showed that low molecular weight heparin (LMWH) was significantly more efficacious than LDUH, with a relative risk reduction of proximal DVT with LMWH of 58%, compared to 30% for LDUH (*P *= 0.01) [521]. Moreover, LMWH was shown to be significantly more efficacious than IPC, with a 1% rate of proximal DVT or pulmonary embolism versus 3% for IPC [522]. More recently, the Prophylaxis for Thromboembolism in Critical Care Trial showed more benefit with LMWH when dalteparin was compared to unfractionated heparin (UFH) in a critical care population; there were similar rates of proximal DVT at about 5%, but the rate of pulmonary embolism was significantly lower with dalteparin (1.3% vs. 2.3% in the UFH group) and a 5% rate of major bleeding [523].

Side effects associated with the use of heparin include heparin-induced thrombocytopenic thrombosis. This effect is seen more frequently with UFH than LMWH. The severity of trauma has been associated with the risk of heparin-induced thrombocytopenia; therefore, the greater the risk, the greater the importance of monitoring platelet counts in trauma patients [524]. In summary, the use of heparin once haemostasis has been achieved is the most efficacious option for trauma patients. In those with a bleeding risk, mechanical methods are preferable. Due to the varied results from trials comparing UFH with LMWH, we do not recommend one over the other. Because LMWHs are mainly excreted renally, unlike UFH, which is excreted through the liver as well, there is risk of accumulation in patients with renal failure; therefore, dose adjustments and/or monitoring should be performed with LMWH according to the manufacturer's instructions.

Contraindications to pharmacological thromboprophylaxis include patients already receiving full-dose anticoagulation, those with significant thrombocytopenia (platelet count <50 × 10^9^/l), an untreated inherited or acquired bleeding disorder, evidence of active bleeding, uncontrolled hypertension (blood pressure >230/120), a lumbar puncture/spinal analgesia expected within the next 12 h or performed within the last 4 h (24 h if traumatic), procedures with a high bleeding risk or a new haemorrhagic stroke.

The use of prophylactic inferior vena cava filters is common; however, no evidence of added benefit when used in combination with pharmacological thromboprophylaxis exists. Pulmonary embolisms still occur despite the presence of a filter, and filters have short and long-term complication rates, are associated with high cost and often provide a false sense of security, delaying the use of effective pharmacological thromboprophylaxis. Furthermore, inferior vena cava filters require a second invasive procedure to remove them.

The optimal timing for the initiation of pharmacological thromboprophylaxis is often difficult to judge. Data from 175,000 critical care admissions showed that the risk of mortality was higher in those who did not receive thromboprophylaxis during the first 24 h [525]. This reflects the concern that those who bleed have a higher rate of venous thromboembolism than those who do not [526].

### VI. Treatment pathway

#### Treatment algorithm

##### Recommendation 35

We recommend that each institution implement an evidence-based treatment algorithm for the bleeding trauma patient. (Grade 1C)

#### Checklists

##### Recommendation 36

We recommend that treatment checklists be used to guide clinical management. (Grade 1B)

#### Quality management

##### Recommendation 37

We recommend that each institution include an assessment of adherence to the institutional algorithm in routine quality management. (Grade 1C)

###### Rationale

The development of a multi-disciplinary evidence-based treatment algorithm for the bleeding trauma patient offers a unique opportunity to create awareness among all involved medical specialities and to improve mutual understanding. The treatment algorithm allows, within the framework of the available evidence, flexibility to accommodate local pre-hospital rescue conditions, locally available diagnostic and therapeutic options and improves the consistency of care. Numerous examples demonstrate the value of a treatment algorithm in improving the care of trauma patients; some also resulted in cost savings [527,528]. Conversely, deviation from treatment pathways increases morbidity and mortality in trauma patients, with a three-fold increased mortality in the subgroup of major deviations [529].

The implementation of our recommendations and adherence to a local treatment algorithm is facilitated by a checklist analogous to the Safe Surgery Initiative [530]. Suggested items that should be included in such a checklist are summarised in Table 4. Trauma treatment training should be an integral part of the implementation of the algorithm.

**Table 4:** Treatment pathway checklist

Treatment phase		Yes	No	N/A	Reason for variance
**Initial assessment and management**					
	Extent of traumatic hemorrhage assessed	□	□	□	
	Patient in shock with identified source of bleeding treated immediately	□	□	□	
	Patient in shock with unidentified source of bleeding sent for further investigation	□	□	□	
	Coagulation, haematocrit, serum lactate, base deficit assessed	□	□	□	
	Antifibrinolytic therapy initiated	□	□	□	
	Patient history of anticoagulant therapy assessed (vitamin K antagonists, antiplatelet agents, oral anticoagulants)	□	□	□	

**Resuscitation**					
	Systolic blood pressure of 80 to 100 mmHg achieved in absence of TBI	□	□	□	
	Measures to achieve normothermia implemented	□	□	□	
	Target Hb level 7 to 9 g/dL achieved	□	□	□	

**Surgical intervention**					
	Abdominal bleeding control achieved	□	□	□	
	Pelvic ring closed and stabilised	□	□	□	
	Peritoneal packing, angiographic embolisation or surgical bleeding control completed in haemodynamically unstable patient	□	□	□	
	Damage control surgery performed in haemodynamically unstable patient	□	□	□	
	Local haemostatic measures applied	□	□	□	
	Thromboprophylactic therapy recommended	□	□	□	

**Coagulation management**					
	Coagulation, haematocrit, serum lactate, base deficit, calcium reassessed	□	□	□	
	Target fibrinogen level 1.5 to 2 g/L achieved	□	□	□	
	Target platelet level achieved	□	□	□	
	Prothrombin complex concentrate administered if indicated due to vitamin-K antagonist or viscoelastic monitoring	□	□	□	

N/A, not applicable.

In addition, adherence to the institutional treatment algorithm should be included as part of routine institutional quality management. Most institutions have established a quality improvement program to assist clinical teams in evaluating their own performance. An audit of adherence to best practice, including feedback and practice change where needed should be included as part of the local implementation of these guidelines. In order to evaluate the quality of care provided to the patient who is bleeding after major trauma, we suggest that the following quality standards be used:

â€¢ Time from injury to the initiation of intervention to stop bleeding (surgery or embolisation) in hypotensive patients who do not respond to initial resuscitation.

â€¢ Time from hospital arrival to availability of a full set of blood results (full blood count, PT, fibrinogen, calcium, viscoelastic testing (if available)).

â€¢ Proportion of patients receiving TXA before leaving the emergency room.

â€¢ Time from hospital arrival to CT scan in bleeding patients without an obvious source of hemorrhage.

â€¢ Damage control surgical techniques are used in accordance with Recommendation 21.

â€¢ Thromboprophylaxis commenced in accordance with Recommendation 34.

Extended post-discharge follow-up times may be required to provide longer-term outcome data, because an increasing percentage of trauma mortality occurs after hospital discharge [531,532]. Approximately 50% of mortality among trauma patients older than 65 years of age occurs between 30 days and 6 months after injury [532].

## Discussion

This guideline for the management of the bleeding trauma patient is based on a critical appraisal of the published literature, a re-appraisal of the recommendations we published three years ago and a consideration of current clinical practice in areas in which randomised clinical trials may never be performed for practical or ethical reasons. In the process of generating this updated version of the guideline, we identified a number of scientific questions that have emerged or were not addressed previously and have developed recommendations to cover these issues. The new and revised recommendations included here reflect newly available evidence, shifts in patient profiles and the consequent adaptation of general clinical practice.

**Figure 2:**
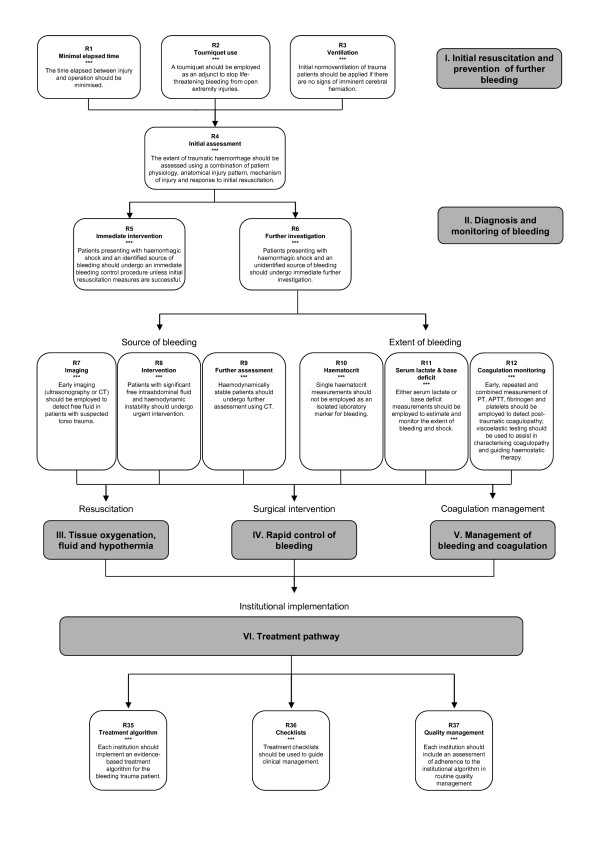
**Flow chart of treatment modalities for the bleeding trauma patient discussed in this guideline (Part 1 of 2)**. APTT, activated partial thromboplastin time; CT, computed tomography; Hb, haemoglobin; PCC, prothrombin complex concentrate; PT, prothrombin time.

**Figure 3:**
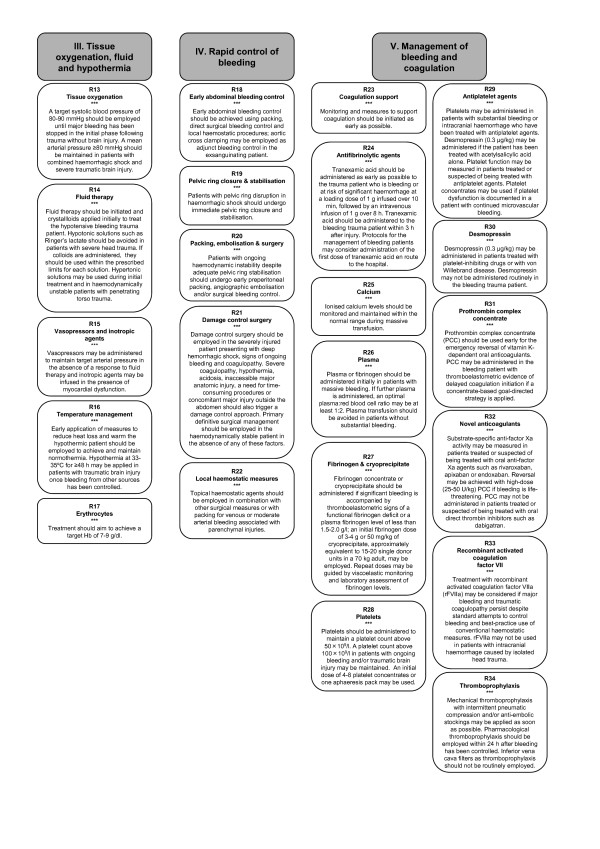
**Flow chart of treatment modalities for the bleeding trauma patient discussed in this guideline (Part 2 of 2)**. APTT, activated partial thromboplastin time; CT, computed tomography; Hb, haemoglobin; PCC, prothrombin complex concentrate; PT, prothrombin time.

All of the recommendations presented here were formulated according to a consensus reached by the author group and the professional societies involved. Figure 2 and Figure 3 graphically summarise the recommendations included in this guideline. We have employed the GRADE [24] hierarchy or evidence to formulate each recommendation because it allows strong recommendations to be supported by weak clinical evidence in areas in which the ideal randomised controlled clinical trials may never be performed. To minimise the bias introduced by individual experts, we employed a nominal group process to develop each recommendation and several rounds of review and discussion to reach an agreement on the questions to be considered and to reach a final consensus on each recommendation. To ensure that the process included input from all of the relevant specialties, the group comprised a multidisciplinary pan-European group of experts, including the active involvement of representatives from five of the most relevant European professional societies.

This version of the guideline includes a new section on the appropriate use of vasopressors and inotropic agents and reflects an awareness of the growing number of patients in the population at large treated with antiplatelet agents and/or oral anticoagulants. As the elderly population grows, clinical practice must adapt to provide optimal care for patients with inherent thromboembolic risk profiles and simultaneously accommodate possible pre-treatment with preventative medications. We continue to concur that both children and elderly adults who have not been pre-treated with anticoagulant or antiplatelet agents should generally be managed in the same manner as the normal adult patient. The current guideline also includes recommendations and a discussion of thromboprophylactic strategies for all patients following traumatic injury.

The most significant addition to this version of the guideline is a new section that discusses the need for every institution to develop, implement and adhere to an evidence-based clinical protocol to manage traumatically injured patients. The author group feels strongly that a comprehensive, multidisciplinary approach to trauma care and mechanisms with which to ensure that established protocols are consistently implemented will ensure a uniform and high standard of care across Europe and beyond. This guideline is a central feature of the STOP the Bleeding Campaign, which aims to reduce the number of patients who die within 24 h after arrival in hospital due to exsanguination by at least 20% within five years. In order to achieve this goal, educational, implementation and compliance control steps must be taken by each institution. These guidelines serve as part of an educational strategy; however, educational steps alone often fail to translate new research results into clinical practice, as has been shown with the introduction of protective lung ventilation [533,534]. One tool with which institutions could measure and compare individual performance and assess the effectiveness of overall treatment would be the establishment of a European trauma database that includes pre-defined quality indicators such as the time required to stop bleeding, 30-day mortality and morbidity. The newly initiated campaign aims to support institutions in the development and implementation of locally adapted protocols, assist in the definition of management bundles and encourage each institution to establish systems with which to assess compliance with the management strategy.

## Conclusions

A multidisciplinary approach to the management of the traumatically injured patient remains the cornerstone of optimal patient care. Each institution needs to develop, implement and adhere to an evidence-based management protocol that has been adapted to local circumstances. As new evidence becomes available, both these clinical practice guidelines and local protocols will need to evolve accordingly.

## Key messages

• Coagulation monitoring and measures to support coagulation should be implemented as early as possible following traumatic injury and used to guide haemostatic therapy.

• A damage control approach to surgical procedures should guide patient management, including closure and stabilisation of pelvic ring disruptions, packing, embolisation and local haemostatic measures.

• This guideline reviews appropriate physiological targets and suggested use and dosing of fluids, blood products and pharmacological agents in the bleeding trauma patient.

• The growing number of older patients requires special attention to appropriately manage the inherent thromboembolic risk profiles and possible pre-treatment with antiplatelet agents and/or oral anticoagulants.

• A multidisciplinary approach to the management of the traumatically injured patient remains the cornerstone of optimal patient care, and each institution needs to develop, implement and adhere to an evidence-based management protocol that has been adapted to local circumstances.

## Abbreviations

ACS: abdominal compartment syndrome; APA: antiplatelet agent; APTT: activated partial thromboplastin time; ARDS: acute respiratory distress syndrome; ATLS: Advanced Trauma Life Support; CT: computed tomography; DDAVP: 1-deamino-8-D-arginine vasopressin; DPL: diagnostic peritoneal lavage; DVT: deep venous thrombosis; FFP: fresh frozen plasma; GCS: Glasgow coma score; GRADE: Grading of Recommendations Assessment: Development and Evaluation; Hb: haemoglobin; Hct: haematocrit; HES: hydroxyethyl starch; ICH: intracranial hemorrhage; ICP: intracranial pressure; ICU: intensive care unit; INR: international normalised ratio; IPC: intermittent pneumatic compression; IQR: interquartile ratio; ISS: Injury Severity Score; IV: intravenous; LDUH: low-dose unfractionated heparin; LMWH: low molecular weight heparin; MCF: maximum clot firmness; MeSH: medical subject heading; MSCT: multi-slice computed tomography; NABIS: H II: National Acute Brain Injury Study: Hypothermia II; NE: norepinephrine; PCC: prothrombin complex concentrate; PEEP: positive end-expiratory pressure; PFA: platelet function analyser; DPT: prothrombin time; RBC: red blood cells; RCT: randomised controlled trial; rFVIIa: recombinant activated coagulation factor VII; TASH: trauma associated severe hemorrhage; TBI: traumatic brain injury; TRALI: transfusion-related acute lung injury; TXA: tranexamic acid; UFH: unfractionated heparin.

## Competing interests

BB has received honoraria for consulting from Novo Nordisk, CSL Behring and Sangart. VC has received honoraria for consulting or lecturing from B. Braun, Fresenius, Novo Nordisk and MSD. He has received research grant funding and institutional support from Charles University in Prague (Czech Republic). TJC has received research grant funding from the National Institute of Health Research and the College of Emergency Medicine. He has received institutional support from the University of Leicester. JD has received institutional support from Assistance Publique Hopitaux de Paris and Paris-Sud University. EFM has received honoraria for consulting from Sangart and CSL Behring. He is member of Medical Advisory Board of Pulsion BJH. DF has received honoraria for consulting or lecturing from Abbott, Sanofi Aventis, Servier and ViforPharma, institutional support from Abbott, Edwards Lifescience, Infomed Fluids, Medtronic, Nycomed, Pfizer, Servier, Siramed and ViforPharma and travel grants from B. Braun, Fresenius Kabi and GlaxoSmithKline.

BJH has received no personal pecunary benefit from pharmaceutical companies, but donated all honoraria from lecturing to charity. She was a joint investigator on a research study funded by Sanofi. BJH does not sit on advisory boards to pharmaceutical companies, but sits on an advisory board for Haemonetics. RK has received honoraria for consulting and lecturing from Eli Lilly and Amgen. MM has received honoraria for consulting or lecturing from Novo Nordisk, CSL Behring and Biotest. He has received research grant funding and institutional support from the Private University Witten-Herdecke (Germany). He has served as a Medical Advisory Board member for CSL Behring. GN has received honoraria for consulting and lecturing from CSL Behring and honoraria for lecturing from Fresenius Kabi. He has received a research grant from Sangart and a research grant (institutional research) from Novo Nordisk.

EN has received honoraria for consulting or lecturing from BIOMET, Pfizer, QRx Pharma, MSD, Grünenthal and Therabel. He has received research grant funding from BMBF, DFG, Else-Kröner Foundation, different societies and has received institutional support from KCI, Pfizer, Mundipharma, BIOMET and Janssen. I YO has received honoraria for consulting or lecturing from LFB and CSL Behring. LR been involved in educational courses on bleeding control supported by Baxter. RR has received honoraria for consulting or lecturing from CSL Behring, Novo Nordisk, Bayer Healthcare and Air Liquide. He has received research grant funding from CSL Behring, Boehringer Ingelheim, Air Liquide, Biotest, Nycomed and Novo Nordisk. AS has no competing interests to declare.

DRS's academic department has received grant support from the Swiss National Science Foundation, Berne, Switzerland (grant numbers: 33CM30_124117 and 406440-131268), the Swiss Society of Anesthesiology and Reanimation (SGAR), Berne, Switzerland (no grant numbers are attributed), the Swiss Foundation for Anesthesia Research, Zurich, Switzerland (no grant numbers are attributed), Bundesprogramm Chancengleichheit, Berne, Switzerland (no grant numbers are attributed), CSL Behring, Berne, Switzerland (no grant numbers are attributed), Vifor SA, Villars-sur-Glâne, Switzerland (no grant numbers are attributed). DRS was the chairman of the ABC Faculty and is a member of the ABC-Trauma Faculty, which both are managed by Physicians World Europe GmbH, Mannheim, Germany and sponsored by unrestricted educational grants from Novo Nordisk Health Care AG, Zurich, Switzerland and CSL Behring GmbH, Marburg, Germany. DRS has received honoraria or travel support for consulting or lecturing from the following companies: Abbott AG, Baar, Switzerland, AMGEN GmbH, Munich, Germany, AstraZeneca AG, Zug, Switzerland, Bayer (Schweiz) AG, Zürich, Switzerland, Baxter AG, Volketswil, Switzerland, Baxter S.p.A., Roma, Italy, B. Braun Melsungen AG, Melsungen, Germany, Boehringer Ingelheim (Schweiz) GmbH, Basel, Switzerland, Bristol-Myers-Squibb, Rueil-Malmaison Cedex, France and Baar, Switzerland, CSL Behring GmbH, Hattersheim am Main, Germany and Berne, Switzerland, Curacyte AG, Munich, Germany, Ethicon Biosurgery, Sommerville, New Jersey, USA, Fresenius SE, Bad Homburg v.d.H., Germany, Galenica AG, Bern, Switzerland (including Vifor SA, Villars-sur-Glâne, Switzerland), GlaxoSmithKline GmbH & Co. KG, Hamburg, Germany, Janssen-Cilag AG, Baar, Switzerland, Janssen-Cilag EMEA, Beerse, Belgium, Merck Sharp & Dohme-Chibret AG, Opfikon-Glattbrugg, Switzerland, Novo Nordisk A/S, Bagsvärd, Denmark, Octapharma AG, Lachen, Switzerland, Organon AG, Pfäffikon/SZ, Switzerland, Oxygen Biotherapeutics, Costa Mesa, CA, Pentapharm GmbH (now tem Innovations GmbH), Munich, Germany, ratiopharm Arzneimittel Vertriebs-GmbH, Vienna, Austria, Roche Pharma (Schweiz) AG, Reinach, Switzerland, Schering-Plough International, Inc., Kenilworth, NJ, USA, Vifor Pharma Deutschland GmbH, Munich, Germany, Vifor Pharma Österreich GmbH, Vienna, Austria, Vifor (International) AG, St. Gallen, Switzerland.

JLV has no competing interests to declare.

The ABC-T European medical education initiative is managed by Physicians World Europe GmbH (Mannheim, Germany) and supported by educational grants from CSL Behring GmbH (Marburg, Germany) and LFB Biomédicaments (Courtaboeuf, France).

## Authors' contributions

All of the authors participated in the formulation of questions to be addressed in the guideline, screening of abstracts and literature, face-to-face and remote consensus-finding processes, drafting, review, revision and approval of the manuscript.

## Authors' information

DRS serves as co-chair of the Advanced Bleeding Care in Trauma (ABC-T) European Medical Education Initiative. VC, TJC, JD and EF-M are members of the ABC-T European Medical Education Initiative faculty. JD represented the European Society of Intensive Care Medicine (ESICM) on the ABC-T Task Force. DF represented the European Society of Anaesthesiology (ESA) on the ABC-T Task Force. RK represented the European Society of Trauma and Emergency Surgery (ESTES) on the ABC-T Task Force. YO represented the European Society of Intensive Care Medicine (ESICM) on the ABC-T Task Force. LR represented the European Society for Emergency Medicine (EuSEM) on the ABC-T Task Force. AS represented the European Shock Society (ESS) on the ABC-T Task Force. RR serves as chair of the ABC-T European Medical Education Initiative.

## Supplementary Material

Additional file 1 MeSH terms and limits applied to address guideline literature queries - 2012. Click here for file

Additional file 2 Additional literature published after the literature search cut-off. Click here for file
