# Supplementary material for: Management of bleeding and coagulopathy following major trauma: an updated European guideline
Source: Crit Care. 2013 Apr 19;17(2):R76. doi: 10.1186/cc12685 (PMC4056078; doi:10.1186/cc12685)
Supplement: Additional file 1 — MeSH terms and limits applied to address guideline literature queries - 2012. [file cc12685-S1.DOCX]

**Additional data file 1**. MeSH terms and limits applied to address guideline literature queries – 2012

| **Question** | | | **Query** | **Limit** |
| --- | --- | --- | --- | --- |
| **I** | **Initial resuscitation and prevention of further bleeding** | | | |
|  | **1** | **Does coagulopathy have an effect on outcome in patients with different types of injury?** | | |
|  |  | “Wounds and Injuries”[MeSH] AND “Blood coagulation disorders”[MeSH] | | Clinical Trial, Meta-Analysis, Practice Guideline, Randomized Controlled Trial, Review, Humans, English, 2009-end |
|  | **2** | **Does control of acid-base balance during the initial resuscitation affect outcome?** | | |
|  |  | "Wounds and Injuries"[MAJR] AND "Acid-Base Equilibrium"[MeSH] | | Humans  English  2009-end |
|  | **3** | **Does the degree of initial bleeding affect coagulopathy?** | | |
|  | **4** | **Does the degree of initial bleeding affect outcome?** | | |
|  |  | “Wounds and Injuries”[MeSH] AND “Hemorrhage”[MeSH] AND “Hemostasis”[MeSH] | | Humans  English  2009-end |
|  | **5** | **Does initial resuscitation with respect to haemostasis have an effect on outcome in patients with different types of injury?** | | |
|  |  | ("Wounds and Injuries"[MeSH] OR "injuries"[Subheading] OR "Craniocerebral Trauma"[MeSH] OR "Trauma Severity Indices"[MeSH] OR "Trauma Centers"[MeSH] OR "Cumulative Trauma Disorders"[MeSH] OR "Cerebrovascular Trauma"[MeSH] OR "Multiple Trauma"[MeSH] OR "Trauma, Nervous System"[MeSH] OR "Head Injuries, Penetrating"[MeSH] OR "Abducens Nerve Injury"[MeSH] OR "Optic Nerve Injuries"[MeSH] OR "Coma, Post-Head Injury"[MeSH] OR "Facial Nerve Injuries"[MeSH] OR "Carotid Artery Injuries"[MeSH] OR "Head Injuries, Closed"[MeSH] OR "Spinal Cord Injuries"[MeSH] OR "Traumatology"[MeSH] OR "Brain Injuries"[MeSH] OR "Emergency Treatment"[MeSH] OR "Emergencies"[MeSH] OR "Critical Care"[MeSH] OR "Emergency Treatment"[MeSH]) AND ("Resuscitation"[MeSH] OR "Resuscitation Orders"[MeSH]) AND ("Hemostatic Techniques"[MeSH] OR "Hemostasis"[MeSH]) | | Humans  English  2009-end |
|  | **6** | **Is wound compression effective in preventing bleeding and coagulopathy?** | | |
|  | a | ("Wounds and Injuries"[MeSH] OR "injuries"[Subheading] OR "Craniocerebral Trauma"[MeSH] OR "Trauma Severity Indices"[MeSH] OR "Trauma Centers"[MeSH] OR "Cumulative Trauma Disorders"[MeSH] OR "Cerebrovascular Trauma"[MeSH] OR "Multiple Trauma"[MeSH] OR "Trauma, Nervous System"[MeSH] OR "Head Injuries, Penetrating"[MeSH] OR "Abducens Nerve Injury"[MeSH] OR "Optic Nerve Injuries"[MeSH] OR "Coma, Post-Head Injury"[MeSH] OR "Facial Nerve Injuries"[MeSH] OR "Carotid Artery Injuries"[MeSH] OR "Head Injuries, Closed"[MeSH] OR "Spinal Cord Injuries"[MeSH] OR "Traumatology"[MeSH] OR "Brain Injuries"[MeSH] OR "Emergency Treatment"[MeSH] OR "Emergencies"[MeSH] OR "Critical Care"[MeSH] OR "Emergency Treatment"[MeSH]) AND ("Resuscitation"[MeSH] OR "Resuscitation Orders"[MeSH]) AND ("Hemostatic Techniques"[MeSH] OR "Hemostasis"[MeSH]) AND ("Bandages"[MeSH] OR "Tourniquets"[Mesh]) | | Humans  English  2009-end |
|  | b | ("Hemostatic Techniques"[MeSH] OR "Hemostasis"[MeSH]) AND ("Bandages"[MeSH] OR "Tourniquets"[Mesh]) | | Humans  English  2009-end |
|  | c | "Tourniquets"[Mesh] | | Humans  English  2009-end |
|  | **7** | **Does the duration of the pre-hospital phase of initial resuscitation have an effect on outcome in patients with haemorrhagic shock?** | | |
|  |  | "Shock, Hemorrhagic"[MeSH] AND ("Emergency Medical Services"[MeSH] OR "Emergencies"[MeSH] OR "Ambulances"[MeSH] OR "Emergency Medical Technicians"[MeSH] OR "Emergency Treatment"[MeSH] OR "Emergency Medical Tags"[MeSH] OR "Emergency Nursing"[MeSH] OR "Emergency Medicine"[MeSH] OR "Emergency Medical Service Communication Systems"[MeSH] OR "Air Ambulances"[MeSH]) AND (pre-hospital OR prehospital) | | Humans  English  2009-end |
|  | **8** | **Does the amount of bleeding and type of injury influence the selection of the hospital to which the trauma patient should be transported?** | | |
|  |  | "Health Care Category"[MAJR] AND "Hemorrhage"[MeSH] AND "Wounds and Injuries"[MeSH] AND ("Emergencies"[MeSH] OR "Emergency Treatment"[MeSH] OR "Emergency Medicine"[MeSH] OR "Emergency Medical Technicians"[MeSH]) | | Humans  English  2009-end |
|  | **9** | **Does the gender of the bleeding trauma patient affect outcome?** | | |
|  | **10** | **Does the age of the bleeding trauma patient affect outcome?** | | |
|  | a | “Wounds and Injuries”[MeSH] AND “Hemorrhage”[MeSH] AND “Hemostasis”[MeSH] AND ("sex"[MeSH Terms] OR "Age Factors"[Mesh]) | | Humans  English |
|  | b | "Wounds and Injuries"[MeSH] AND "Blood coagulation disorders"[MeSH] AND ("sex"[MeSH Terms] OR "Female"[Mesh] OR "Male"[Mesh] OR "Age Factors"[MeSH] OR "Aged"[MeSH] OR "Geriatrics"[Mesh]) | | Humans  English  2009-end |
|  | **11** | **Is there a specific coagulation disorder associated with traumatic injury?** | | |
|  |  | “Wounds and Injuries”[MeSH] AND “Hemorrhage”[MeSH] AND “Hemostasis”[MeSH] AND "Blood Coagulation Disorders"[Mesh] | | Humans  English |
|  |  |  |  |  |
| **II** | **Diagnosis and monitoring of bleeding** | | | |
|  | **1** | **Is there evidence to support a correlation between the mechanism of injury and the risk of bleeding?** | | |
|  |  | “Wounds and Injuries”[MAJR] AND “Hemorrhage”[MeSH] AND correlation[All Fields] | | Humans  English  2009-end |
|  | **2** | **Which clinical signs are most appropriate to detect the patient who is actively bleeding?** | | |
|  |  | "Wounds and Injuries"[MeSH] AND "Hemorrhage"[MeSH] AND ("Emergencies"[MeSH] OR "Emergency Treatment"[MeSH] OR "Emergency Medicine"[MeSH] OR "Emergency Medical Technicians"[MeSH]) AND ("Diagnosis"[MeSH] OR "Signs and Symptoms"[MeSH]) | | Humans  English  2009-end |
|  | **3** | **Which laboratory parameters (biochemical tests) are most appropriate to detect the patient who is actively bleeding?** | | |
|  |  | "Wounds and Injuries" [MeSH] AND "Hemorrhage" [MeSH] AND ("Clinical Chemistry Tests"[MeSH] OR "Monitoring, Physiologic" [MeSH] OR "Chemistry, Clinical"[MeSH] OR "Biological Markers"[MeSH] OR "Intercellular Signaling Peptides and Proteins"[MeSH]) | | Humans  English  2009-end |
|  | **4** | **Which imaging diagnostic tools are most appropriate to detect the patient who is actively bleeding?** | | |
|  |  | "Wounds and injuries"[MeSH] AND "Hemorrhage"[MeSH] AND "Diagnostic Imaging"[MeSH] AND ("Emergencies"[MeSH] OR "Emergency Treatment"[MeSH] OR "Emergency Medicine"[MeSH] OR "Emergency Medical Technicians"[MeSH]) | | Humans  English  2009-end |
|  | **5** | **Is there evidence to support the use of a specific score to assess the extent of bleeding?** | | |
|  | **6** | **Is the ATLS shock classification still a valid classification system?** | | |
|  |  | "Hemorrhage"[MeSH] AND "Trauma Severity Indices"[MeSH] AND "Wounds and Injuries"[MeSH] | | Humans  English  2009-end |
|  | **7** | **Which coagulation monitoring tools are most appropriate to detect the patient who is actively bleeding?** | |  |
|  |  | ("Wounds and Injuries/diagnosis"[MAJR] OR "Hemorrhage/diagnosis"[MAJR] OR "Blood Coagulation Disorders/diagnosis"[MAJR] OR "Blood Coagulation Factors/analysis"[MAJR] OR "Blood Coagulation Factors/diagnostic use"[MAJR]) AND ("Point-of-Care Systems"[Mesh] OR "Thrombelastography"[Mesh]) | | Humans  English  2009-end |
|  |  | ("Wounds and Injuries/diagnosis"[MAJR] AND "Hemorrhage/diagnosis"[MAJR]) AND ("Tomography, X-Ray"[Mesh] OR "Tomography, X-Ray Computed"[Mesh] OR "Multidetector Computed Tomography"[Mesh] OR "Ultrasonography"[Mesh] OR "Peritoneal Lavage"[Mesh]) | | Humans  English  2009-end |
|  | **8** | **Which coagulation monitoring tools are most appropriate to detect post-traumatic coagulopathy?** | | |
|  |  | "Wounds and Injuries"[MeSH] AND ("Hemorrhage"[Mesh] OR "Disseminated Intravascular Coagulation"[Mesh]) AND ("Emergencies"[MeSH] OR "Emergency Treatment"[MeSH] OR "Emergency Medicine"[MeSH] OR "Emergency Medical Technicians"[MeSH]) AND ("Diagnosis"[MeSH] OR "Monitoring, Physiologic"[MeSH] OR "Point-of-Care Systems"[Mesh] OR "Thrombelastography"[Mesh] OR "Predictive Value of Tests"[Mesh]) | | Humans  English  2009-end |
|  | **9** | **Does the type of monitoring tool used have an impact on outcome?** | | |
|  |  | "Wounds and Injuries"[MeSH] AND ("Hemorrhage"[Mesh] OR "Disseminated Intravascular Coagulation"[Mesh]) AND ("Emergencies"[MeSH] OR "Emergency Treatment"[MeSH] OR "Emergency Medicine"[MeSH] OR "Emergency Medical Technicians"[MeSH]) AND "Outcome Assessment (Health Care)"[Mesh] AND ("Pathological Conditions, Signs and Symptoms"[Mesh] OR "Diagnosis"[MeSH] OR "Point-of-Care Systems"[Mesh] OR "Thrombelastography"[Mesh] OR "Predictive Value of Tests"[Mesh]) | | Humans  English  2009-end |
|  | **10** | **Using which coagulation monitoring tools can the effectiveness of therapeutic measures be monitored?** | | |
|  | **11** | **Is there any advantage to thrombelastometry performed at the bedside?** | | |
|  | **12** | **Is there any advantage to thrombelastometry performed in the central laboratory?** | | |
|  |  | "Wounds and Injuries"[MeSH] AND (("Blood Coagulation"[Mesh] AND "Blood Coagulation Disorders"[Mesh]) OR "Hemorrhage "[MeSH] OR “Shock, Hemorrhagic”[MeSH]) AND "Treatment Outcome"[Mesh] AND "Therapeutics"[Mesh] AND ("Emergencies"[MeSH] OR "Emergency Treatment"[MeSH] OR "Emergency Medicine"[MeSH] OR "Emergency Medical Technicians"[MeSH]) AND ("Diagnosis"[MeSH] OR “Monitoring, Physiologic” [MeSH] OR "Point-of-Care Systems"[Mesh] OR "Thrombelastography"[Mesh]) | | Humans  English  2009-end |
|  | **13** | **Do early detection or diagnostic assessment of coagulation status influence outcome?** | | |
|  |  | "Wounds and Injuries"[MeSH] AND ("Hemorrhage"[Mesh] OR "Disseminated Intravascular Coagulation"[Mesh]) AND ("Emergencies"[MeSH] OR "Emergency Treatment"[MeSH] OR "Emergency Medicine"[MeSH] OR "Emergency Medical Technicians"[MeSH]) AND ("Diagnosis"[MeSH] OR "Monitoring, Physiologic"[MeSH] OR "Point-of-Care Systems"[Mesh] OR "Thrombelastography"[Mesh]) AND ("Early Medical Intervention"[Mesh] OR "Early Diagnosis"[Mesh]) | | Humans  English |
|  |  |  |  |  |
| **III** | **Tissue oxygenation, type of fluid and hypothermia** | | | |
|  | **1** | **What is the corridor for the haematocrit to be achieved for adequate tissue oxygenation?** | | |
|  |  | "Hematocrit"[MeSH] AND ("Oxygen Consumption"[MeSH] OR "Blood Gas Monitoring, Transcutaneous"[MeSH] OR "Blood Gas Analysis"[MeSH] OR "Gases"[MeSH] OR "Cell Respiration"[MeSH] OR "Blood Substitutes"[MeSH] OR "Blood Chemical Analysis"[MeSH] OR "Respiratory Function Tests"[MeSH] OR "Oximetry"[MeSH] OR "Blood Pressure"[MeSH] OR "Venous Pressure"[MeSH] OR "Hypotension"[MeSH]) AND ("Emergencies"[MeSH] OR "Emergency Treatment"[MeSH] OR "Emergency Medicine"[MeSH] OR "Emergency Medical Technicians"[MeSH] OR "Wounds and Injuries"[MeSH] OR "Craniocerebral Trauma"[MeSH] OR "Cerebrovascular Trauma"[MeSH] OR "Multiple Trauma"[MeSH] OR "Trauma, Nervous System"[MeSH] OR "Head Injuries, Penetrating"[MeSH] OR "Carotid Artery Injuries"[MeSH] OR "Head Injuries, Closed"[MeSH] OR "Spinal Cord Injuries"[MeSH] OR "Traumatology"[MeSH] OR "Brain Injuries"[MeSH]) | | Humans  English  2009-end |
|  | **2** | **How should volume loading be managed?** | | |
|  |  | ("Blood Substitutes"[MeSH] OR "Fluorocarbons"[MeSH] OR "Plasma Substitutes"[MeSH] OR "Fluorocarbons"[MeSH] OR "Plasma Substitutes"[MeSH] OR "Fluid Therapy"[MeSH] OR "Rehydration Solutions"[MeSH] OR "Solutions"[MeSH] OR "Colloids"[MeSH] OR "Sodium Chloride"[MeSH] OR "Saline Solution, Hypertonic"[MeSH]) AND ("Wounds and Injuries"[MeSH] OR "Craniocerebral Trauma"[MeSH] OR "Cerebrovascular Trauma"[MeSH] OR "Multiple Trauma"[MeSH] OR "Trauma, Nervous System"[MeSH] OR "Head Injuries, Penetrating"[MeSH] OR "Carotid Artery Injuries"[MeSH] OR "Head Injuries, Closed"[MeSH] OR "Spinal Cord Injuries"[MeSH] OR "Traumatology"[MeSH] OR "Brain Injuries"[MeSH]) AND ("Infusions, Parenteral"[MeSH] OR "Infusions, Intravenous"[MeSH]) | | Humans  English  2009-end |
|  | **3** | **Does the blood pressure achieved during initial resuscitation influence morbidity or outcome in the trauma patient?** | | |
|  |  | ("Blood Pressure Determination"[MeSH] OR "Blood Pressure"[MeSH] OR "Blood Pressure Monitoring, Ambulatory"[MeSH] OR "Hypertension"[MeSH] OR "Venous Pressure"[MeSH] OR "Hypotension"[MeSH]) AND ("Wounds and Injuries"[MeSH] OR "Craniocerebral Trauma"[MeSH] OR "Cerebrovascular Trauma"[MeSH] OR "Multiple Trauma"[MeSH] OR "Trauma, Nervous System"[MeSH] OR "Head Injuries, Penetrating"[MeSH] OR "Carotid Artery Injuries"[MeSH] OR "Head Injuries, Closed"[MeSH] OR "Spinal Cord Injuries"[MeSH] OR "Traumatology"[MeSH] OR "Brain Injuries"[MeSH]) AND ("Emergencies"[MeSh] OR "Emergency Treatment"[MeSH] OR "Emergency Medicine"[MeSh] OR "Emergency Medical Technicians"[MeSH]) | | Humans  English  2009-end |
|  | **4** | **Does the type of fluid used for initial resuscitation influence morbidity or outcome in the trauma patient?** | | |
|  |  | ("Blood Substitutes"[MeSH] OR "Fluorocarbons"[MeSH] OR "Plasma Substitutes"[MeSH] OR "Fluorocarbons"[MeSH] OR "Plasma Substitutes"[MeSH] OR "Fluid Therapy"[MeSH] OR "Rehydration Solutions"[MeSH] OR "Solutions"[MeSH] OR "Colloids"[MeSH] OR "Sodium Chloride"[MeSH] OR "Saline Solution, Hypertonic"[MeSH]) AND ("Wounds and Injuries"[MeSH] OR "Craniocerebral Trauma"[MeSH] OR "Cerebrovascular Trauma"[MeSH] OR "Multiple Trauma"[MeSH] OR "Trauma, Nervous System"[MeSH] OR "Head Injuries, Penetrating"[MeSH] OR "Carotid Artery Injuries"[MeSH] OR "Head Injuries, Closed"[MeSH] OR "Spinal Cord Injuries"[MeSH] OR "Traumatology"[MeSH] OR "Brain Injuries"[MeSH]) AND ("Emergencies"[MeSH] OR "Emergency Treatment"[MeSH] OR "Emergency Medicine"[MeSH] OR "Emergency Medical Technicians"[MeSH]) | | Humans  English  2009-end |
|  | **5** | **Does controlled mild hypothermia (34°C) affect outcome or morbidity in the trauma patient compared to normothermia?** | | |
|  |  | ("Hypothermia"[MeSH] OR "Gastric Hypothermia"[MeSH] OR "Hypothermia, Induced"[MeSH] OR "Circulatory Arrest, Deep Hypothermia Induced"[MeSH]) AND "Outcome and Process Assessment (Health Care)"[MeSH] AND ("Wounds and Injuries"[MeSH] OR "injuries"[Subheading] OR "Craniocerebral Trauma"[MeSH] OR "Trauma Severity Indices"[MeSH] OR "Trauma Centers"[MeSH] OR "Cumulative Trauma Disorders"[MeSH] OR "Cerebrovascular Trauma"[MeSH] OR "Multiple Trauma"[MeSH] OR "Trauma, Nervous System"[MeSH] OR "Head Injuries, Penetrating"[MeSH] OR "Abducens Nerve Injury"[MeSH] OR "Optic Nerve Injuries"[MeSH] OR "Coma, Post-Head Injury"[MeSH] OR "Facial Nerve Injuries"[MeSH] OR "Carotid Artery Injuries"[MeSH] OR "Head Injuries, Closed"[MeSH] OR "Spinal Cord Injuries"[MeSH] OR "Traumatology"[MeSH] OR "Brain Injuries"[MeSH]) | | Humans  English  2009-end |
|  | **6** | **Which types of colloids are appropriate for use under which circumstances in the bleeding trauma patient?** | | |
|  |  | ("Wounds and Injuries"[MeSH] OR "Craniocerebral Trauma"[MeSH] OR "Cerebrovascular Trauma"[MeSH] OR "Multiple Trauma"[MeSH] OR "Trauma, Nervous System"[MeSH] OR "Head Injuries, Penetrating"[MeSH] OR "Carotid Artery Injuries"[MeSH] OR "Head Injuries, Closed"[MeSH] OR "Spinal Cord Injuries"[MeSH] OR "Traumatology"[MeSH] OR "Brain Injuries"[MeSH]) AND ("Emergencies"[MeSH] OR "Emergency Treatment"[MeSH] OR "Emergency Medicine"[MeSH] OR "Emergency Medical Technicians"[MeSH]) AND "Colloids"[MeSH] | | Humans  English |
|  | **7** | **Which types of crystalloids are appropriate for use under which circumstances in the bleeding trauma patient?** | | |
|  | **8** | **Is the use of Ringer’s lactate appropriate in patients with severe head injury?** | | |
|  |  | ("Wounds and Injuries"[MeSH] OR "Craniocerebral Trauma"[MeSH] OR "Cerebrovascular Trauma"[MeSH] OR "Multiple Trauma"[MeSH] OR "Trauma, Nervous System"[MeSH] OR "Head Injuries, Penetrating"[MeSH] OR "Carotid Artery Injuries"[MeSH] OR "Head Injuries, Closed"[MeSH] OR "Spinal Cord Injuries"[MeSH] OR "Traumatology"[MeSH] OR "Brain Injuries"[MeSH]) AND ("Emergencies"[MeSH] OR "Emergency Treatment"[MeSH] OR "Emergency Medicine"[MeSH] OR "Emergency Medical Technicians"[MeSH]) AND ("Sodium Chloride"[MeSH] OR "Saline Solution, Hypertonic"[MeSH]) | | Humans  English |
|  | **9** | **Is there an appropriate use of vasopressors or inotropic agents in the bleeding trauma patient?** | | |
|  | **10** | **Are vasopressors or inotropic agents harmful in the bleeding trauma patient?** | | |
|  |  | ("Emergencies"[MAJR] OR "Emergency Treatment"[ MAJR] OR "Emergency Medicine"[ MAJR] OR "Emergency Medical Technicians"[ MAJR] OR "Wounds and Injuries"[ MAJR] OR "Craniocerebral Trauma"[ MAJR] OR "Cerebrovascular Trauma"[ MAJR] OR "Multiple Trauma"[ MAJR] OR "Trauma, Nervous System"[ MAJR] OR "Head Injuries, Penetrating"[ MAJR] OR "Carotid Artery Injuries"[ MAJR] OR "Head Injuries, Closed"[ MAJR] OR "Spinal Cord Injuries"[ MAJR] OR "Traumatology"[ MAJR] OR "Brain Injuries"[ MAJR]) AND ("Vasoconstrictor Agents"[Mesh] OR "Cardiotonic Agents"[MeSH]) | | Humans  English  10 years |
|  |  |  |  |  |
| **IV** | **Rapid control of bleeding** | | | |
|  | **1** | **Can the mechanism of injury (e.g. blunt vs. penetrating trauma) be used as a determinant for deciding which patients in haemorrhagic shock are candidates for surgical bleeding control?** | | |
|  |  |  | “Wounds and Injuries”[MAJR] AND “Hemorrhage”[MeSH] AND “Shock, Hemorrhagic”[MeSH] AND (“Emergencies”[MeSH] OR “Emergency Treatment”[MeSH] OR “Emergency Medicine”[MeSH] OR “Emergency Medical Technicians”[MeSH]) | Humans  English  2009-end |
|  | **2** | **Does angiographic embolisation improve the outcome of patients with haemorrhagic shock and pelvic ring disruption?** | | |
|  |  |  | “Embolization, Therapeutic”[MeSH] AND (“Shock, Hemorrhagic”[MeSH] OR “Fractures”[MeSH]) | Humans  English  2009-end |
|  | **3** | **What are the characteristics of patients with free intraabdominal fluid according to FAST in whom secondary diagnostics (i.e. CT scan) can be safely performed?** | | |
|  |  |  | “Wounds and Injuries”[MeSH] AND (“Diagnostic Imaging”[MeSH] OR “Ultrasonography”[MeSH]) AND “Ascitic Fluid”[MeSH] | Humans  English  2009-end |
|  | **4** | **What characterises the patient in heamorrhagic shock in whom in whom immediate aortic cross-clamping is warranted?** | | |
|  |  |  | “Wounds and Injuries”[MeSH] AND (“Diagnostic Imaging”[MeSH] OR “Ultrasonography”[MeSH]) AND “Ascitic Fluid”[MeSH] “Shock, Hemorrhagic”[MeSH] AND (“Angioscopy”[MeSH] OR “Heart, Assist Devices”[MeSH] OR “Cardiovascular Surgical Procedures”[MeSH] OR “Surgical Procedures Minimally Invasive”[MeSH]) | Humans  English  2009-end |
|  | **5** | **Does the elapsed time from admission to OR influence outcome for trauma patients who need emergency surgery?** | | |
|  |  |  | ("Emergency Medical Services"[MeSH] OR "Emergencies"[MeSH] OR "Emergency Treatment"[MeSH] OR "Critical Care"[MeSH]) AND "Hemorrhage"[MeSH] AND "Outcome and Process Assessment (Health Care)"[MeSH] AND ("Wounds and Injuries"[MeSH] OR "injuries"[Subheading] OR "Craniocerebral Trauma"[MeSH] OR "Trauma Severity Indices"[MeSH] OR "Trauma Centers"[MeSH] OR "Cumulative Trauma Disorders"[MeSH] OR "Cerebrovascular Trauma"[MeSH] OR "Multiple Trauma"[MeSH] OR "Trauma, Nervous System"[MeSH] OR "Head Injuries, Penetrating"[MeSH] OR "Abducens Nerve Injury"[MeSH] OR "Optic Nerve Injuries"[MeSH] OR "Coma, Post-Head Injury"[MeSH] OR "Facial Nerve Injuries"[MeSH] OR "Carotid Artery Injuries"[MeSH] OR "Head Injuries, Closed"[MeSH] OR "Spinal Cord Injuries"[MeSH] OR "Traumatology"[MeSH] OR "Brain Injuries"[MeSH]) | Humans  English  2009-end |
|  | **6** | **Does urgent surgery to control haemorrhage improve the outcome of patients with haemorrhagic shock and pelvic ring disruption?** | | |
|  |  |  | "Hip Fractures"[MeSH] AND "Shock, Hemorrhagic"[MeSH] | Humans  English  2009-end |
|  | **7** | **What characterises the patient with free intraabdominal fluid according to FAST who requires immediate laparotomy?** | | |
|  |  |  | "Ascitic Fluid"[MeSH] AND "Laparotomy"[MeSH] AND ("Wounds and Injuries"[MeSH] OR "injuries"[Subheading] OR "Craniocerebral Trauma"[MeSH] OR "Trauma Severity Indices"[MeSH] OR "Trauma Centers"[MeSH] OR "Cumulative Trauma Disorders"[MeSH] OR "Cerebrovascular Trauma"[MeSH] OR "Multiple Trauma"[MeSH] OR "Trauma, Nervous System"[MeSH] OR "Head Injuries, Penetrating"[MeSH] OR "Abducens Nerve Injury"[MeSH] OR "Optic Nerve Injuries"[MeSH] OR "Coma, Post-Head Injury"[MeSH] OR "Facial Nerve Injuries"[MeSH] OR "Carotid Artery Injuries"[MeSH] OR "Head Injuries, Closed"[MeSH] OR "Spinal Cord Injuries"[MeSH] OR "Traumatology"[MeSH] OR "Brain Injuries"[MeSH]) | Humans  English  2009-end |
|  | **8** | **What characterises the patient in haemorrhagic shock in whom packing is warranted?** | | |
|  |  |  | “Wounds and Injuries”[MeSH] AND (“Ascitic Fluid”[MeSH] OR “Shock, Hemorrhagic”[MeSH] OR “Hemorrhage”[MeSH]) AND "Abdominal Injuries"[Mesh] AND ("Hemostasis, Surgical"[Mesh] OR "Hemostatic Techniques"[Mesh] OR "Surgical Procedures, Minimally Invasive"[Mesh]) | Humans  English  2009-end |
|  | **9** | **What characterises the patient in haemorrhagic shock in whom use of a tourniquet is warranted?** | | |
|  |  |  | “Wounds and Injuries”[MeSH] AND (“Hemorrhage”[MeSH] OR “Shock, Hemorrhagic”[MeSH]) AND "Tourniquets"[Mesh] | Humans  English  2009-end |
|  | **10** | **Does the use of local haemostatic agents improve outcome of patients with haemorrhagic shock?** | | |
|  |  |  | “Wounds and Injuries”[MeSH] AND (“Hemorrhage”[MeSH] OR “Shock, Hemorrhagic”[MeSH]) AND "Hemostatic Techniques"[Mesh] AND ("Fibrin Tissue Adhesive"[Mesh] OR "Fibrin Foam"[Mesh] OR "Gelatin Sponge, Absorbable"[Mesh] OR "Thrombin"[Mesh] OR "Thromboplastin"[Mesh]) | Humans  English  2009-end |
|  | **11** | **Does urgent surgery to prevent further bleeding improve outcome for patients with haemorrhagic shock?** | | |
|  |  |  | “Wounds and Injuries”[MeSH] AND (“Hemorrhage”[MeSH] OR “Shock, Hemorrhagic”[MeSH]) AND "Hemostasis, Surgical"[Mesh] | Humans  English  2009-end |
|  |  |  |  |  |
| **V** | **Management of bleeding and coagulation** | | | |
|  |  | ***Red Cells*** | |  |
|  | **1** | **What is the indication for the use of RBC in bleeding after trauma?** | | |
|  | **2** | **Does administration of RBCs affect clinical outcome in bleeding patients?** | | |
|  | **3** | **What are the risks of giving RBCs?** | | |
|  | **4** | **How many RBCs are enough?** | | |
|  |  | ("Wounds and Injuries"[MeSH] OR "Craniocerebral Trauma"[MeSH] OR "Cerebrovascular Trauma"[MeSH] OR "Multiple Trauma"[MeSH] OR "Trauma, Nervous System"[MeSH] OR "Head Injuries, Penetrating"[MeSH] OR "Carotid Artery Injuries"[MeSH] OR "Head Injuries, Closed"[MeSH] OR "Spinal Cord Injuries"[MeSH] OR "Traumatology"[MeSH] OR "Brain Injuries"[MeSH]) AND ("Erythrocytes"[MeSH] OR "Erythrocyte Transfusion"[MeSH]) | | Humans  English  2009-end |
|  | **5** | **What is the effect of RBC transfusion on microcirculation?** | | |
|  |  | ("Wounds and Injuries"[MeSH] OR "Craniocerebral Trauma"[MeSH] OR "Cerebrovascular Trauma"[MeSH] OR "Multiple Trauma"[MeSH] OR "Trauma, Nervous System"[MeSH] OR "Head Injuries, Penetrating"[MeSH] OR "Carotid Artery Injuries"[MeSH] OR "Head Injuries, Closed"[MeSH] OR "Spinal Cord Injuries"[MeSH] OR "Traumatology"[MeSH] OR "Brain Injuries"[MeSH]) AND ("Erythrocytes"[MeSH] OR "Erythrocyte Transfusion"[MeSH]) AND "Microcirculation"[Mesh] | | Humans  English |
|  | **6** | **Does the duration of RBC storage affect clinical outcome?** | | |
|  |  | ("Wounds and Injuries"[MeSH] OR "Craniocerebral Trauma"[MeSH] OR "Cerebrovascular Trauma"[MeSH] OR "Multiple Trauma"[MeSH] OR "Trauma, Nervous System"[MeSH] OR "Head Injuries, Penetrating"[MeSH] OR "Carotid Artery Injuries"[MeSH] OR "Head Injuries, Closed"[MeSH] OR "Spinal Cord Injuries"[MeSH] OR "Traumatology"[MeSH] OR "Brain Injuries"[MeSH]) AND ("Erythrocytes"[MeSH] OR "Erythrocyte Transfusion"[MeSH]) AND "Blood Preservation"[Mesh] | | Humans  English |
|  | **7** | **What is the appropriate use of epoetin in post-traumatic anaemia?** | | |
|  |  | ("Wounds and Injuries"[MeSH] OR "Craniocerebral Trauma"[MeSH] OR "Cerebrovascular Trauma"[MeSH] OR "Multiple Trauma"[MeSH] OR "Trauma, Nervous System"[MeSH] OR "Head Injuries, Penetrating"[MeSH] OR "Carotid Artery Injuries"[MeSH] OR "Head Injuries, Closed"[MeSH] OR "Spinal Cord Injuries"[MeSH] OR "Traumatology"[MeSH] OR "Brain Injuries"[MeSH]) AND ("Erythrocytes"[MeSH] OR "Erythrocyte Transfusion"[MeSH] OR "Anemia"[Mesh]) AND "Erythropoietin"[Mesh] | | Humans  English |
|  | **8** | **Is there a role for iron therapy in the treatment of post-traumatic anaemia?** | | |
|  |  | ("Wounds and Injuries"[MeSH] OR "Craniocerebral Trauma"[MeSH] OR "Cerebrovascular Trauma"[MeSH] OR "Multiple Trauma"[MeSH] OR "Trauma, Nervous System"[MeSH] OR "Head Injuries, Penetrating"[MeSH] OR "Carotid Artery Injuries"[MeSH] OR "Head Injuries, Closed"[MeSH] OR "Spinal Cord Injuries"[MeSH] OR "Traumatology"[MeSH] OR "Brain Injuries"[MeSH]) AND ("Erythrocytes"[MeSH] OR "Erythrocyte Transfusion"[MeSH] OR "Anemia"[Mesh]) AND "Iron"[Mesh] | | Humans  English |
|  | **9** | **Which massive transfusion guidelines have been published?** | | |
|  | **10** | **Which massive transfusion guidelines are used in clinical practice: institutional, national, international?** | | |
|  | **11** | **How uniform are massive transfusion guidelines?** | | |
|  | **12** | **Are massive transfusion guidelines adhered to in clinical practice?** | | |
|  |  | ("Wounds and Injuries"[MeSH] OR "Craniocerebral Trauma"[MeSH] OR "Cerebrovascular Trauma"[MeSH] OR "Multiple Trauma"[MeSH] OR "Trauma, Nervous System"[MeSH] OR "Head Injuries, Penetrating"[MeSH] OR "Carotid Artery Injuries"[MeSH] OR "Head Injuries, Closed"[MeSH] OR "Spinal Cord Injuries"[MeSH] OR "Traumatology"[MeSH] OR "Brain Injuries"[MeSH]) AND ("Erythrocytes"[MeSH] OR "Erythrocyte Transfusion"[MeSH]) AND AND ("Guideline" [Publication Type] OR "Evidence-Based Emergency Medicine"[Mesh] OR "Evidence-Based Practice"[Mesh] OR "Guideline Adherence"[Mesh] OR "Evidence-Based Medicine"[Mesh]) | | Humans  English |
|  |  | ***FFP*** | | |
|  | **13** | **What is the indication for giving FFP to patients bleeding after trauma?** | | |
|  | **14** | **Does administration of FFP reduce the severity of bleeding in patients with acquired coagulation factor deficits?** | | |
|  | **15** | **Does administration of FFP affect clinical outcome in bleeding patients with acquired coagulation factor deficits?** | | |
|  | **16** | **What are the risks of administration of FFP?** | | |
|  | **17** | **What is the recommended dose of FFP to be given?** | | |
|  |  | ("Wounds and Injuries"[MeSH] OR "Craniocerebral Trauma"[MeSH] OR "Cerebrovascular Trauma"[MeSH] OR "Multiple Trauma"[MeSH] OR "Trauma, Nervous System"[MeSH] OR "Head Injuries, Penetrating"[MeSH] OR "Carotid Artery Injuries"[MeSH] OR "Head Injuries, Closed"[MeSH] OR "Spinal Cord Injuries"[MeSH] OR "Traumatology"[MeSH] OR "Brain Injuries"[MeSH]) AND ("Plasma"[MeSH] OR "Plasma Exchange"[MeSH]) | | Humans  English  2009-end |
|  | **18** | **What is the recommended dose of FFP relative to the dose of RBCs administered?** | | |
|  | **19** | **Does the relative dose of FFP:RBCs have an impact on outcome?** | | |
|  | **20** | **Is there any benefit to a 1:1:1 transfusion regimen?** | | |
|  | **21** | **Is there any benefit to goal-directed coagulation management using factor replacement?** | | |
|  |  | ("Emergencies"[MeSH] OR "Emergency Treatment"[MeSH] OR "Emergency Medicine"[MeSH] OR "Emergency Medical Technicians"[MeSH] OR "Wounds and Injuries"[MeSH] OR "Craniocerebral Trauma"[MeSH] OR "Cerebrovascular Trauma"[MeSH] OR "Multiple Trauma"[MeSH] OR "Trauma, Nervous System"[MeSH] OR "Head Injuries, Penetrating"[MeSH] OR "Carotid Artery Injuries"[MeSH] OR "Head Injuries, Closed"[MeSH] OR "Spinal Cord Injuries"[MeSH] OR "Traumatology"[MeSH] OR "Brain Injuries"[MeSH]) AND ("Erythrocytes"[MeSH] OR "Erythrocyte Transfusion"[MeSH]) AND ("Plasma"[MeSH] OR "Plasma Exchange"[MeSH] OR "Blood Platelets"[MeSH] OR "Thrombocytopenia"[MeSH]) | | Humans  English |
|  |  | ***Platelets*** | | |
|  | **22** | **What is the indication for the use of platelets in bleeding after trauma?** | | |
|  | **23** | **Does administration of platelets reduce the severity of bleeding in patients with thrombocytopenia?** | | |
|  | **24** | **Does administration of platelets affect clinical outcome in bleeding patients with thrombocytopenia?** | | |
|  | **25** | **What are the risks of giving platelets?** | | |
|  |  | ("Blood Platelets"[MeSH] OR "Thrombocytopenia"[MeSH]) AND ("Wounds and Injuries"[MeSH] OR "Craniocerebral Trauma"[MeSH] OR "Cerebrovascular Trauma"[MeSH] OR "Multiple Trauma"[MeSH] OR "Trauma, Nervous System"[MeSH] OR "Head Injuries, Penetrating"[MeSH] OR "Carotid Artery Injuries"[MeSH] OR "Head Injuries, Closed"[MeSH] OR "Spinal Cord Injuries"[MeSH] OR "Traumatology"[MeSH] OR "Brain Injuries"[MeSH]) | | Humans  English  2009-end |
|  | **26** | **Does the duration of platelet storage affect clinical outcome?** | | |
|  |  | ("Wounds and Injuries"[MeSH] OR "Craniocerebral Trauma"[MeSH] OR "Cerebrovascular Trauma"[MeSH] OR "Multiple Trauma"[MeSH] OR "Trauma, Nervous System"[MeSH] OR "Head Injuries, Penetrating"[MeSH] OR "Carotid Artery Injuries"[MeSH] OR "Head Injuries, Closed"[MeSH] OR "Spinal Cord Injuries"[MeSH] OR "Traumatology"[MeSH] OR "Brain Injuries"[MeSH]) AND ("Blood Platelets"[MeSH] OR "Thrombocytopenia"[MeSH]) AND "Blood Preservation"[Mesh] | | Humans  English |
|  |  | ***Fibrinogen and Cryoprecipitate*** | |  |
|  | **27** | **What are the indications for fibrinogen or cryoprecipitate?** | | |
|  | **28** | **What is the recommended cryoprecipitate dose in bleeding patients?** | | |
|  | **29** | **Have fibrinogen or cryoprecipitate been shown to be efficacious in bleeding patients?** | | |
|  | **30** | **What are the risks of using fibrinogen or cryoprecipitate in bleeding patients?** | | |
|  |  | ("Wounds and Injuries"[MeSH] OR "Craniocerebral Trauma"[MeSH] OR "Cerebrovascular Trauma"[MeSH] OR "Multiple Trauma"[MeSH] OR "Trauma, Nervous System"[MeSH] OR "Head Injuries, Penetrating"[MeSH] OR "Carotid Artery Injuries"[MeSH] OR "Head Injuries, Closed"[MeSH] OR "Spinal Cord Injuries"[MeSH] OR "Traumatology"[MeSH] OR "Brain Injuries"[MeSH]) AND ("Fibrinogen"[MeSH] OR "cryoprecipitate coagulum"[Substance Name] OR "Nour-Eldin fraction"[Substance Name]) | | Humans  English  2009-end |
|  | **31** | **What is the recommended target fibrinogen level?** | | |
|  | **32** | **Which coagulation monitoring tools are most appropriate to detect fibrinogen level?** | | |
|  |  | ("Emergencies"[MeSH] OR "Emergency Treatment"[MeSH] OR "Emergency Medicine"[MeSH] OR "Emergency Medical Technicians"[MeSH] OR "Wounds and Injuries"[MeSH] OR "Craniocerebral Trauma"[MeSH] OR "Cerebrovascular Trauma"[MeSH] OR "Multiple Trauma"[MeSH] OR "Trauma, Nervous System"[MeSH] OR "Head Injuries, Penetrating"[MeSH] OR "Carotid Artery Injuries"[MeSH] OR "Head Injuries, Closed"[MeSH] OR "Spinal Cord Injuries"[MeSH] OR "Traumatology"[MeSH] OR "Brain Injuries"[MeSH]) AND ("Fibrinogen"[MeSH] OR "cryoprecipitate coagulum"[Substance Name] OR "Nour-Eldin fraction"[Substance Name]) AND ("administration and dosage "[Subheading] OR "Monitoring, Physiologic"[Mesh] OR "Biomarkers, Pharmacological"[Mesh] OR "Drug Monitoring"[Mesh] OR "Point-of-Care Systems"[Mesh] OR "Thrombelastography"[Mesh]) | | Humans  English  2009-end |
|  |  | ***Anti-fibrinolytic agents*** | |  |
|  | **33** | **What are the indications for anti-fibrinolytics in a bleeding patient?** | | |
|  | **34** | **What are the recommended doses of anti-fibrinolytic agents in bleeding patients?** | | |
|  | **35** | **Have anti-fibrinolytics been shown to be efficacious?** | | |
|  | **36** | **What are the risks of using anti-fibrinolytics in bleeding patients?** | | |
|  | **37** | **Which anti-fibrinolytic(s) are preferable based on comparative data and risk benefit analysis?** | | |
|  | **38** | **Which antifibrinolytic agent(s) are appropriate for use in the bleeding trauma patient?** | | |
|  | **39** | **Is the use of antifibrinolytic agents appropriate in all cases of suspected significant bleeding?** | | |
|  | **40** | **Does the timing of antifibrinolytic treatment affect outcome?** | | |
|  |  | "Antifibrinolytic Agents"[MeSH] AND ("Hemorrhage"[MeSH] OR "Brain Stem Hemorrhage, Traumatic"[MeSH] OR "Subarachnoid Hemorrhage"[MeSH] OR "Cerebral Hemorrhage"[MeSH] OR "Cerebral Hemorrhage, Traumatic"[MeSH] OR "Subarachnoid Hemorrhage, Traumatic"[MeSH] OR "Brain Hemorrhage, Traumatic"[MeSH] OR "Intracranial Hemorrhage, Traumatic"[MeSH]) NOT "Aprotinin"[Mesh] | | Humans  English  2009-end |
|  | **41** | **Is the use of antifibrinolytic agents cost effective?** | |  |
|  |  | ("Hemorrhage"[MeSH] OR "Brain Stem Hemorrhage, Traumatic"[MeSH] OR "Subarachnoid Hemorrhage"[MeSH] OR "Cerebral Hemorrhage"[MeSH] OR "Cerebral Hemorrhage, Traumatic"[MeSH] OR "Subarachnoid Hemorrhage, Traumatic"[MeSH] OR "Brain Hemorrhage, Traumatic"[MeSH] OR "Intracranial Hemorrhage, Traumatic"[MeSH]) AND “Antifibrinolytic Agents"[MeSH] NOT "Aprotinin"[Mesh] AND ("Costs and Cost Analysis"[Mesh] OR "Cost-Benefit Analysis"[Mesh] OR "Cost of Illness"[Mesh] OR "Cost Savings"[Mesh] OR "Health Care Costs"[Mesh] OR "Hospital Costs"[Mesh] OR "Drug Costs"[Mesh]) | | Humans  English |
|  |  | ***Coagulation factor concentrates*** | |  |
|  | **42** | **What are the indications for the use of coagulation factor concentrates in a bleeding patient?** | | |
|  | **43** | **What are the recommended doses of coagulation factor concentrates in a bleeding patient?** | | |
|  | **44** | **Have coagulation factor concentrates been shown to be efficacious?** | | |
|  | **45** | **What are the risks of using coagulation factor concentrates in bleeding patients?** | | |
|  |  | ("Emergencies"[MeSH] OR "Emergency Treatment"[MeSH] OR "Emergency Medicine"[MeSH] OR "Emergency Medical Technicians"[MeSH] OR "Wounds and Injuries"[MeSH] OR "Craniocerebral Trauma"[MeSH] OR "Cerebrovascular Trauma"[MeSH] OR "Multiple Trauma"[MeSH] OR "Trauma, Nervous System"[MeSH] OR "Head Injuries, Penetrating"[MeSH] OR "Carotid Artery Injuries"[MeSH] OR "Head Injuries, Closed"[MeSH] OR "Spinal Cord Injuries"[MeSH] OR "Traumatology"[MeSH] OR "Brain Injuries"[MeSH]) AND ("Factor XIII"[Mesh] OR "recombinant FVIIa" [Supplementary Concept]) | | Humans  English  2009-end |
|  |  | ***Prothrombin complex concentrates*** | |  |
|  | **46** | **What is the indication for PCCs in bleeding patients?** | | |
|  | **47** | **What is the recommended dose of PCCs in bleeding patients?** | | |
|  | **48** | **Have PCCs been shown to be efficacious in bleeding patients?** | | |
|  | **49** | **What are the risks of using PCCs in bleeding patients?** | | |
|  | **50** | **Is there comparative data on the transfusion of PCCs compared with FFP in bleeding patients?** | | |
|  |  | ("Wounds and Injuries"[MeSH] OR "Craniocerebral Trauma"[MeSH] OR "Cerebrovascular Trauma"[MeSH] OR "Multiple Trauma"[MeSH] OR "Trauma, Nervous System"[MeSH] OR "Head Injuries, Penetrating"[MeSH] OR "Carotid Artery Injuries"[MeSH] OR "Head Injuries, Closed"[MeSH] OR "Spinal Cord Injuries"[MeSH] OR "Traumatology"[MeSH] OR "Brain Injuries"[MeSH]) AND ("Thromboplastin"[MeSH] OR "Partial Thromboplastin Time"[MeSH] OR "Factor XI"[MeSH] OR "thromboplastin apoprotein, human"[Substance Name] OR "prothrombinase complex"[Substance Name] OR "Factor IXa"[MeSH] OR "Prothrombin Time"[MeSH] OR "Factor VII"[MeSH] OR "Factor IX"[MeSH] OR "prothrombin complex concentrates"[Substance Name]) | | Humans  English  2009-end |
|  |  | ***Desmopressin*** | |  |
|  | **51** | **What are the indications for the use of desmopressin in a bleeding patient?** | | |
|  | **52** | **What is the recommended dose of desmopressin in a bleeding patient?** | | |
|  | **53** | **Has desmopressin been shown to be efficacious?** | | |
|  | **54** | **What are the risks of using desmopressin in bleeding patients?** | | |
|  |  | ("Emergencies"[MeSH] OR "Emergency Treatment"[MeSH] OR "Emergency Medicine"[MeSH] OR "Emergency Medical Technicians"[MeSH] OR "Wounds and Injuries"[MeSH] OR "Craniocerebral Trauma"[MeSH] OR "Cerebrovascular Trauma"[MeSH] OR "Multiple Trauma"[MeSH] OR "Trauma, Nervous System"[MeSH] OR "Head Injuries, Penetrating"[MeSH] OR "Carotid Artery Injuries"[MeSH] OR "Head Injuries, Closed"[MeSH] OR "Spinal Cord Injuries"[MeSH] OR "Traumatology"[MeSH] OR "Brain Injuries"[MeSH]) AND "Deamino Arginine Vasopressin"[Mesh] | | Humans  English  2009-end |
|  |  | ***Calcium*** | | |
|  | **55** | **Under which circumstances should ionised calcium levels be monitored?** | | |
|  |  | ("Emergencies"[MAJR] OR "Emergency Treatment"[ MAJR] OR "Emergency Medicine"[ MAJR] OR "Emergency Medical Technicians"[ MAJR] OR "Wounds and Injuries"[ MAJR] OR "Craniocerebral Trauma"[ MAJR] OR "Cerebrovascular Trauma"[ MAJR] OR "Multiple Trauma"[ MAJR] OR "Trauma, Nervous System"[ MAJR] OR "Head Injuries, Penetrating"[ MAJR] OR "Carotid Artery Injuries"[ MAJR] OR "Head Injuries, Closed"[ MAJR] OR "Spinal Cord Injuries"[ MAJR] OR "Traumatology"[ MAJR] OR "Brain Injuries"[ MAJR]) AND "Calcium"[MAJR] | | Humans  English  2009-end |
|  | **56** | **What is the appropriate use of calcium chloride in the bleeding trauma patient?** | | |
|  |  | ("Emergencies"[MAJR] OR "Emergency Treatment"[ MAJR] OR "Emergency Medicine"[ MAJR] OR "Emergency Medical Technicians"[ MAJR] OR "Wounds and Injuries"[ MAJR] OR "Craniocerebral Trauma"[ MAJR] OR "Cerebrovascular Trauma"[ MAJR] OR "Multiple Trauma"[ MAJR] OR "Trauma, Nervous System"[ MAJR] OR "Head Injuries, Penetrating"[ MAJR] OR "Carotid Artery Injuries"[ MAJR] OR "Head Injuries, Closed"[ MAJR] OR "Spinal Cord Injuries"[ MAJR] OR "Traumatology"[ MAJR] OR "Brain Injuries"[ MAJR]) AND "Calcium/therapeutic use"[MAJR] | | Humans  English  2009-end |
|  |  | ***Anti-platelet agents*** | | |
|  | **57** | **Does pre-medication with anti-platelet agents affect clinical outcome in bleeding patients with thrombocytopenia?** | | |
|  |  | ("Wounds and Injuries"[MeSH] OR "Craniocerebral Trauma"[MeSH] OR "Cerebrovascular Trauma"[MeSH] OR "Multiple Trauma"[MeSH] OR "Trauma, Nervous System"[MeSH] OR "Head Injuries, Penetrating"[MeSH] OR "Carotid Artery Injuries"[MeSH] OR "Head Injuries, Closed"[MeSH] OR "Spinal Cord Injuries"[MeSH] OR "Traumatology"[MeSH] OR "Brain Injuries"[MeSH]) AND "Platelet Aggregation Inhibitors"[Mesh] | | Humans  English  2009-end |
|  | **58** | **What is the appropriate use of platelets in the treatment of patients pre-treated with anti-platelet agents?** | |  |
|  |  | ("Emergencies"[MAJR] OR "Emergency Treatment"[ MAJR] OR "Emergency Medicine"[ MAJR] OR "Emergency Medical Technicians"[ MAJR] OR "Wounds and Injuries"[ MAJR] OR "Craniocerebral Trauma"[ MAJR] OR "Cerebrovascular Trauma"[ MAJR] OR "Multiple Trauma"[ MAJR] OR "Trauma, Nervous System"[ MAJR] OR "Head Injuries, Penetrating"[ MAJR] OR "Carotid Artery Injuries"[ MAJR] OR "Head Injuries, Closed"[ MAJR] OR "Spinal Cord Injuries"[ MAJR] OR "Traumatology"[ MAJR] OR "Brain Injuries"[ MAJR]) AND "Platelet Aggregation Inhibitors"[Mesh] AND ("Therapeutics"[Mesh] OR "Emergency Treatment"[Mesh] OR "Blood Platelets"[MeSH]) | | Humans  English |
|  |  | ***Oral anticoagulants*** | |  |
|  | **59** | **Does pre-injury anticoagulant medication influence outcome?** | |  |
|  |  | ("Emergencies"[MAJR] OR "Emergency Treatment"[ MAJR] OR "Emergency Medicine"[ MAJR] OR "Emergency Medical Technicians"[ MAJR] OR "Wounds and Injuries"[ MAJR] OR "Craniocerebral Trauma"[ MAJR] OR "Cerebrovascular Trauma"[ MAJR] OR "Multiple Trauma"[ MAJR] OR "Trauma, Nervous System"[ MAJR] OR "Head Injuries, Penetrating"[ MAJR] OR "Carotid Artery Injuries"[ MAJR] OR "Head Injuries, Closed"[ MAJR] OR "Spinal Cord Injuries"[ MAJR] OR "Traumatology"[ MAJR] OR "Brain Injuries"[ MAJR]) AND ("Outcome Assessment (Health Care)"[Mesh] OR "Treatment Outcome"[Mesh] OR "Outcome and Process Assessment (Health Care)"[Mesh]) AND "Anticoagulants"[Mesh] | | Humans  English |
|  | **60** | **How can drug-induced coagulation disorders be detected and diagnosed?** | | |
|  |  | ("Emergencies"[MAJR] OR "Emergency Treatment"[ MAJR] OR "Emergency Medicine"[ MAJR] OR "Emergency Medical Technicians"[ MAJR] OR "Wounds and Injuries"[ MAJR] OR "Craniocerebral Trauma"[ MAJR] OR "Cerebrovascular Trauma"[ MAJR] OR "Multiple Trauma"[ MAJR] OR "Trauma, Nervous System"[ MAJR] OR "Head Injuries, Penetrating"[ MAJR] OR "Carotid Artery Injuries"[ MAJR] OR "Head Injuries, Closed"[ MAJR] OR "Spinal Cord Injuries"[ MAJR] OR "Traumatology"[ MAJR] OR "Brain Injuries"[ MAJR]) AND ("Diagnosis"[Mesh] OR "Early Diagnosis"[Mesh]) AND "Blood Coagulation Disorders"[Mesh] AND "Anticoagulants"[Mesh] | | Humans  English |
|  | **61** | **How should drug-induced coagulopathies be managed?** | |  |
|  |  | ("Emergencies"[MAJR] OR "Emergency Treatment"[ MAJR] OR "Emergency Medicine"[ MAJR] OR "Emergency Medical Technicians"[ MAJR] OR "Wounds and Injuries"[ MAJR] OR "Craniocerebral Trauma"[ MAJR] OR "Cerebrovascular Trauma"[ MAJR] OR "Multiple Trauma"[ MAJR] OR "Trauma, Nervous System"[ MAJR] OR "Head Injuries, Penetrating"[ MAJR] OR "Carotid Artery Injuries"[ MAJR] OR "Head Injuries, Closed"[ MAJR] OR "Spinal Cord Injuries"[ MAJR] OR "Traumatology"[ MAJR] OR "Brain Injuries"[ MAJR]) AND ("Blood Coagulation Disorders"[Mesh] OR "Anticoagulants"[Mesh]) AND ("Therapeutics"[Mesh] OR "Emergency Treatment"[Mesh]) | | Humans  English  10 years |
|  |  | ***Antithrombotic management*** | |  |
|  | **62** | **What type of antithrombotic therapy is appropriate following traumatic injury?** | | |
|  | **63** | **How can the effect of antithrombotic agents be antagonised if necessary to support coagulation?** | | |
|  |  | ("Emergencies"[MAJR] OR "Emergency Treatment"[ MAJR] OR "Emergency Medicine"[ MAJR] OR "Emergency Medical Technicians"[ MAJR] OR "Wounds and Injuries"[ MAJR] OR "Craniocerebral Trauma"[ MAJR] OR "Cerebrovascular Trauma"[ MAJR] OR "Multiple Trauma"[ MAJR] OR "Trauma, Nervous System"[ MAJR] OR "Head Injuries, Penetrating"[ MAJR] OR "Carotid Artery Injuries"[ MAJR] OR "Head Injuries, Closed"[ MAJR] OR "Spinal Cord Injuries"[ MAJR] OR "Traumatology"[ MAJR] OR "Brain Injuries"[ MAJR]) AND ("Thrombosis/prevention and control"[Mesh] OR "Venous Thrombosis/prevention and control"[Mesh] OR "Pulmonary Embolism/prevention and control"[Mesh]) | | Humans  English  10 years |
|  |  |  | |  |
