# Supplementary material for: Management of bleeding and coagulopathy following major trauma: an updated European guideline
Source: Crit Care. 2013 Apr 19;17(2):R76. doi: 10.1186/cc12685 (PMC4056078; doi:10.1186/cc12685)
Supplement: Additional file 2 — Additional literature published after the literature search cut-off. [file cc12685-S2.DOCX]

This list comprises literature citations that were published after the literature search cut-off for the guideline document, therefore these publications were not selected according to a comprehensive search strategy, but represent work with sufficient relevance to the guideline that inclusion was requested by one or more of the endorsing professional societies as part of the guideline review and endorsement process.

1. Escolar G, Arellano-Rodrigo E, J.C. R, et al.: **Reversal of apixaban-induced alterations of hemostasis by different coagulation factor concentrates: studies in vitro with circulating human blood**. 2012.

2. Harr JN, Moore EE, Ghasabyan A, Chin TL, Sauaia A, Banerjee A, Silliman CC: **Functional fibrinogen assay indicates that fibrinogen is critical in correcting abnormal clot strength following trauma**. *Shock* 2013, **39**(1):45-49.

3. Holcomb JB, Minei KM, Scerbo ML, Radwan ZA, Wade CE, Kozar RA, Gill BS, Albarado R, McNutt MK, Khan S *et al*: **Admission rapid thrombelastography can replace conventional coagulation tests in the emergency department: experience with 1974 consecutive trauma patients**. *Annals of surgery* 2012, **256**(3):476-486.

4. Marlu R, Hodaj E, Paris A, Albaladejo P, Crackowski JL, Pernod G: **Effect of non-specific reversal agents on anticoagulant activity of dabigatran and rivaroxaban: a randomised crossover ex vivo study in healthy volunteers**. *Thrombosis and haemostasis* 2012, **108**(2):217-224.

5. Rahe-Meyer N, Solomon C, Hanke A, Schmidt DS, Knoerzer D, Hochleitner G, Sorensen B, Hagl C, Pichlmaier M: **Effects of Fibrinogen Concentrate as First-line Therapy during Major Aortic Replacement Surgery: A Randomized, Placebo-controlled Trial**. *Anesthesiology* 2013, **118**(1):40-50.

6. Weber CF, Gorlinger K, Meininger D, Herrmann E, Bingold T, Moritz A, Cohn LH, Zacharowski K: **Point-of-care testing: a prospective, randomized clinical trial of efficacy in coagulopathic cardiac surgery patients**. *Anesthesiology* 2012, **117**(3):531-547.
